# Differences Between Household Income from Surveys and Registers and How These Affect the Poverty Headcount: Evidence from the Austrian SILC

**DOI:** 10.1007/s11205-017-1672-7

**Published:** 2017-06-12

**Authors:** Stefan Angel, Richard Heuberger, Nadja Lamei

**Affiliations:** 1 Institute for Social Policy, WU Vienna University of Economics and Business, Welthandelsplatz 1, 1020 Vienna, Austria; 2 Statistics Austria, Guglgasse 13, 1110 Vienna, Austria

**Keywords:** Register data, Poverty, Income measurement, EU-SILC

## Abstract

We take advantage of the fact that for the Austrian SILC 2008–2011, two data sources are available in parallel for the same households: register-based and survey-based income data. Thus, we aim to explain which households tend to under- or over-report their household income by estimating multinomial logit and OLS models with covariates referring to the interview situation, employment status and socio-demographic household characteristics. Furthermore, we analyze source-specific differences in the distribution of household income and how these differences affect aggregate poverty indicators based on household income. The analysis reveals an increase in the cross-sectional poverty rates for 2008–2011 and the longitudinal poverty rate if register data rather than survey data are used. These changes in the poverty rate are mainly driven by differences in employment income rather than sampling weights and other income components. Regression results show a pattern of mean-reverting errors when comparing household income between the two data sources. Furthermore, differences between data sources for both under-reporting and over-reporting slightly decrease with the number of panel waves in which a household participated. Among the other variables analyzed that are related to the interview situation (mode, proxy, interview month), only the number of proxy interviews was (weakly) positively correlated with the difference between data sources, although this outcome was not robust over different model specifications.

**Electronic supplementary material:**

The online version of this article (doi:10.1007/s11205-017-1672-7) contains supplementary material, which is available to authorized users.

## Introduction and Review of the Literature

The policy relevance of social indicators has risen with the latest financial and economic crisis. They were awarded a prominent status in European politics with the European Commission’s Europe 2020-target for social inclusion (2010) and before that the Laeken indicator set on social inclusion (2001). The European Community Statistics on Income and Living Conditions (EU-SILC) are one of the pillars of social statistics in the European Statistical System and the most relevant household survey at the European level in the field of household income, living standards and poverty. Several indicators of social inclusion, amongst them the Europe 2020-target for the “risk of poverty or social exclusion”, are calculated annually on the basis of this source.

Being so highly recognized, those indicators are expected to fulfil high statistical standards concerning reliability, validity and comparability (both over time and between countries). The evaluation of measurement error in this context is therefore crucial. In our paper, we focus on the measurement of household income in EU-SILC and investigate differences between data collected using surveys and data collected from registers. For this purpose, we take advantage of the fact, that for the Austrian EU-SILC of 2008–2011, both register- and survey-based income data are available for the same observational units.^1^ Using the differences in these measurements for households at the micro level, we aim to provide explanations for changes in different income-based poverty indicators by investigating the underlying changes in the distribution of household income as a consequence of using register data. First, by estimating multinomial logit and linear models with covariates referring to the income and employment structure, the interview situation (e.g. CATI vs. CAPI) and other household characteristics, we try to explain whether certain types of households tend to under- or over-report their household income when asked via the survey method. Second, we ask which component (income type, weighting) contributes most to the change in the poverty measurement if register data are used instead of survey data.

### Differences Between Register and Survey Data, Measurement Error and Its Impact on Poverty

The identification of data errors requires by definition some a point of reference to judge the accuracy of the information. In most cases, administrative data are proclaimed to be the benchmark. Bound et al. (2001) distinguish between micro-level and macro-level validation studies for assessing measurement error. Micro-level validation studies usually define measurement error as the difference between the value recorded in administrative records and the value observed in the survey. Macro-level validation studies, in contrast, compare population parameters, such as income inequality or the sum of earnings, derived from the survey to official reports based on administrative records or to estimates obtained from a comparable survey. Existing studies on measurement error are mostly done for the US population and with a focus on personal or market income.

Mean-reverting errors with low earnings inflated and high earnings underreported are a common finding in such studies (Bound et al. 2001; Gottschalk and Huynh 2010; Kim and Tamborini 2012, 2014). Income volatility and income structure also matter: based on a survey sample for a developing country, Akke (2011) found that prior earnings volatility strongly affects measurement error in the current period. Moreover, there is evidence for a positive correlation between measurement error and the number of different income sources in the household (Moore et al. 2000).

Besides income-related variables, studies have also shown the importance of the survey duration and survey mode. In longitudinal studies, panel participants’ responses may increasingly begin to differ from their initial responses to the same survey questions due to learning effects in answering a complex questionnaire and/or by an improved personal relationship between respondent and (the same) interviewer (Sikkel and Hoogendoorn 2008; Chadi 2013). Such effects have been found for questions on life satisfaction (Frick et al. 2006; Landua 1992) and for subjective mental health (Wooden and Li 2014). For income, however, a longer participation in a panel does not necessarily result in higher accuracy. Measurement errors for income are usually found to be positively serially correlated in such studies for the US population (Pischke 1995; Bound and Krueger 1991). Whether this also applies for representative survey data for a population sample in a European country will be investigated in our paper.

Mode effects refer to the type of interaction between interviewer and interviewee. Existing studies focus on differences between CATI and CAPI and on the relevance of proxy interviews for income measurement error. The literature has shown that respondents to CATI are more likely to present socially desirable responses (Beland and St-Pierre 2007; Groves et al. 2009; Holbrook et al. 2003). A study for Austria found that telephone interviews lead to a larger downward bias concerning income inequality (Fessler et al. 2013). For proxy interviews, however, a more ambivalent picture emerges (Brown et al. 2001; Tourangeau et al. 2000). On the one hand, proxy interviews may enhance data quality because there is less social desirability pressure and thus a lower likelihood of mean-reverting errors. On the other hand, income of other household members can easily be overlooked due to recall error or interview fatigue. Some (and mostly older) studies have found only little proxy bias in earnings (Bound and Krueger 1991), whereas more recent studies show that proxy interviews bias earnings downwards (Reynolds and Wenger 2012) and their effect also interacts with demographic variables (Tamborini and Kim 2013).

Furthermore, differences in income inequality are observed when survey and register data are compared: Gottschalk and Huynh (2010) discuss the implications of measurement error in surveys on earnings income inequality. By matching the US Survey of Income and Program Participation to tax data, they find that income inequality is 20% higher in the register data. Based on a random sample from the Danish population, Kreiner et al. (2013) compare a one-shot recall question on total personal income (employment income, pension income, social transfers) with the corresponding tax records of the respondents. The authors find a lower mean and a lager spread for the survey measure.

A smaller number of studies are concerned with total household income and the consequences of using register data instead of survey data for the calculation of household income and poverty indicators. The studies available also differ in their validation methodology. In sum, the measurement error of income has been shown to affect cross-sectional poverty rates (Nordberg et al. 2001; Figari et al. 2012), poverty dynamics (Rendtel et al. 1998; McGarry 1995; Breen and Moisio 2004; Worts et al. 2010) and statistical relationships of poverty indicators with other variables (Lohmann 2011). Nordberg et al. (2001) found that income estimates derived from administrative records are quite reliable and generally higher than surveyed income, except for very low register incomes. They interpreted the differences observed as being mainly due to measurement errors in the interview data. Their results showed that survey data produced higher inequality and poverty estimates than register data. Lohmann (2011) makes use of between-country differences concerning data sources for income variables (register or survey data). Results show that the degree of consistency between earnings and employment status (i.e. no earnings reported if the status is non-working) is on average lower in register countries; this also impacts on the poverty rate conditional on activity status in some countries. The author concludes that the relationship between employment status and poverty status also depends on the data collection approach used. Figari et al. (2012) compare empirical estimates of income distribution and poverty rates based on microsimulation methods with observed survey-data-based estimates. The authors use simulated estimates in their model in accordance with prevailing rules on liability and eligibility in four European countries. On the one hand, their results show that poverty rates, defined as the number of people with equalized incomes less than 60% of the national median, which use reported data are slightly higher than those calculated using simulated incomes. On the other hand, there was an overlap of 75% for both approaches.

### Characteristics of Register Data and Implementation in EU-SILC

Assuming register data to be a less error-prone source for validating survey questions on income, however, may not always be justified (Abowd and Stinson 2013; Kapteyn and Ypma 2007) and depends on the context of data production. Since administrative registers are not initially built to answer certain research questions, they should not be expected to provide perfect statistical data (United Nations 2007; Wallgren and Wallgren 2007; Zhang 2011). Abowd and Stinson (2013) identify three potential causes for deviations from survey data which must not be confounded with different levels of measurement error: a) definitional differences between survey and register data—like taxable income relevant for a wage-tax register versus actual disposable income from a standard-of-living perspective; b) errors in administrative data itself (e.g. coverage issues and updating intervals) and c) and mistakes in the matching process of multiple data sources.

In the European Statistical System, common definitions and methods have been agreed upon in order to facilitate the comparability of poverty indicators and income between countries. EU-SILC^2^ comprises several variables of personal and household income components and is conducted in all 28 member countries plus several more.^3^ In cooperation with the National Statistical Institutes (NSI), Eurostat aims to maximize the comparability of indicators across the participating countries through the output harmonization of variables (i.e. providing/developing explicit conceptual definitions of what to measure, namely so-called “target variables”, as opposed to specifications of how to measure them) and agreements on various methodological aspects like sampling, weighting and precision requirements. However, whereas detailed rules for the content of variables and the construction of those indicators exist, the source of income data—amongst other parameters—is up to the Member States. As a consequence, some countries mainly use official registers, whereas other countries mostly (have to) rely on survey data to fill specific income components. Thus, the heterogeneity of the data sources is something of an obstacle to their comparability, though it may lead to a good overall level of data quality in the outcome indicators.

When EU-SILC started in 2004, only few countries were using registers; but nowadays ever more Member States are making the step towards integrating register data into their SILC data collections. Studies investigating the impact of register use on measurement error are therefore vital. Törmälehto (2013) draws four main conclusions for the context of EU-SILC^4^:

1. Integrating register data in a data collection may affect multiple phases of a survey process: sampling and weighting (as new information from registers can be used e.g. to design the sample), non-response analysis, calibration of weights, survey designs (as the potential for dropping questions from the survey may alter the whole “flow”), processing and quality control, imputation, dissemination and documentation.
2. It is challenging to generalise about quality of registers in a cross-national context.
3. There is a lot of variation concerning the particular data sources for specific variables. Register data may originate from survey-like data collections (e.g. self-administered questionnaires) but also from entirely electronic exchanges of administrative data.
4. The combined use of survey and register data affects the total survey error (Groves et al. 2009), and expands the traditional survey error sources to those related to registers. To explain this, Törmälehto (2013) also cites Zhang (2011), who proposed an addition to Groves’ Total Surveys Error model. While Groves’ ideas were designed for the context of (sampled) survey data, Zhang (2011) further develops and applies them to error sources associated with register data (e.g. problems of conceptualization, measurement, and accuracy). Zhang proposes a “two-phase life cycle of integrated statistical micro data” where the first phase concerns the data from each single source, and the second the integration of data from different sources. Register data could be used as a benchmark against which survey data could be compared to estimate the magnitude and predictors of measurement error in a given country. However, it should not be expected that register data themselves are not prone to (other) sources of error and that the combination of register and survey data leads to perfect statistical data—on the contrary: “At the present stage, there is still clearly a lack of statistical theories for assessing the quality of such register-based statistics.” (Zhang 2011: 446). In sum, there could be more sources of error when using register data, but usually the expectation is to have a lower total error due to fewer measurement errors.

### Effects of Register Data Use in EU-SILC: A Comparative Perspective

Some countries have a longer history of register data use than others, mostly for legal and administrative reasons: Denmark, Finland, Island, Netherlands, Norway, Sweden and Slovenia are those that started with administrative data in SILC right away (i.e. from 2004/05). Then there are those who joined in more recently: Italy gradually since 2004, France since 2008, Austria since 2012, and Spain since 2013. Although the “old” register countries encountered the same challenges,^5^ we focus on those countries that have made the transition from survey income in more recent years and therefore have SILC waves with different income sources to compare.

In Spain, the new methodology, where register and survey income information is combined, is considered a more comprehensive method of collecting income in lower and in higher parts of the distribution (Méndez 2015). Income levels are significantly higher than when using the survey approach but inequality indicators, like the risk of poverty, remain similar. Similarly, the French experience (Burricand 2013) showed that the change in methodology did not have a significant impact on the poverty rate, while other inequality indicators increased. Differences between the two income sources—registers versus surveys—were more important in the extremes of the distribution than in the mid-range, and for some income components (pensions) than others (wages). In Italy (Consolini and Donatiello 2013), the inclusion of register data produced a substantial increase in the estimate of average income among self-employed earners, while the increase for employees was less pronounced. At the same time, the use of a mixed data-collection strategy versus survey data only resulted in a substantial decrease in the risk of poverty and Gini coefficient. Only about half of all persons were at risk of poverty according to both methodologies; the others had a different status with each methodology.

### Conclusions Drawn from the Literature Review

To sum up, the prevailing literature in the field highlights the following problems related to measurement error in income: (a) errors explained by data collection methods (e.g. type of question—yearly vs. current, simple vs. complex; source for income variables—register or survey or any combination of both); (b) problems caused by panel design and relevant for measuring poverty dynamics correctly; and (c) challenges concerning cross-country comparisons. Furthermore, two main conclusions can be derived from existing SILC studies and similar surveys: (1) the effect of register data is generally more visible in the lower and upper extremes of the household income distributions and varies for different income components; (2) the effect of register data use on income inequality and poverty indicators varies between countries.

We add to the literature in several aspects. All of the studies on household income and poverty indicators discussed above use either microsimulation or some variant of Markov modelling to capture measurement error. In this paper, survey data is directly validated against register data at a micro level. Moreover, the consequences of deviations for estimating poverty indicators are investigated. The focus of the analysis lies on equivalised household income. Additionally, studies that report on situations where consent from sampled individuals is necessary to link a survey with register data—as in the US—do not apply in our case, as giving or withdrawing consent introduces a further burden and potential bias. Seeking respondents’ consent is not legally required in Austria for a voluntary survey like SILC. This allows for a more complete comparison of survey and register data across the income distribution and socio-demographic groups. This article thus aims to pave the way to a better understanding of the specifics for Austria and to contribute to a more comprehensive picture in EU-SILC and other large European surveys.

The remainder of the article is structured as follows: Sect. 2 describes the development and specifics of register use for SILC in Austria as well as the context of the re-calculation of household incomes for 2008–2011. It then goes on to illustrate the household income concept and its components. Section 3 describes how the analysis addresses the main research questions. The fourth section is divided into two parts. The first part illustrates the effects of the data switch on the aggregate poverty rate and the underlying statics of the distribution of household income. In the second part, the results of both cross-sectional (for 2010) and longitudinal (2008–2011) regression models for the observed income differences are discussed. The robustness of the main results is further evaluated against different model specifications and statistical tests. The outcomes of these tests are summarized in the fifth section. Section 6 concludes and describes limitations of the current study and suggestions for further research.

## Data

### Register Use in the Austrian EU-SILC

SILC Austria was launched in 2003. At that time, income components were exclusively collected through surveys (CAPI, voluntary participation). Since 2008 CATI interviews have been used for the panel part of the survey. Survey data for particular income components were first substituted by register data in 2012 (Heuberger et al. 2013). The main reasons for gradually switching to register data were quality and response burden considerations.^6^ Together with the Federal Ministry for Labour, Social Affairs and Consumer Protection, Statistics Austria decided to recalculate and revise income data for 2008–2011 using administrative registers. One main target was to shift the break in time series of the EU 2020 Social Inclusion Indicators further back to the baseline year 2008, resulting in parallel data for the same respondents for 2008–2011. In this paper, we compare the Austrian EU-SILC data from 2008 to 2011 before and after that revision. The sample size ranges between 13,621 and 14,085 individuals nested in around 5700–6100 households in these years.

Austrian law stipulates that the linkage of personal micro data from surveys with registers be done using an anonymized personal identifier (*bPK*). However, unlike in many other countries including the USA, it is not necessary to seek consent of the interviewee for the linkage procedure.^7^ In order to identify individuals in the administrative datasets, the personal identifier of people covered by the survey is required. Usually, this information is collected as part of the sampling procedure. The share of identifiers found for the total population in the survey varies over time but generally decreases the farther back the survey year is. For the years analyzed here it ranges between 96% (2008) and 99% (2011). However, there are always individuals who turn out to be actual household members but are not covered by the sampling frame (mainly because they are not officially registered at a particular household’s address). Their linkage key is missing ex ante and must be retrieved by a procedure involving the Federal Ministry for the Interior. For 2008–2011 missing keys most often occur among younger people, persons living in Vienna^8^ (capital) and persons with non-Austrian citizenship. With the exception of EU-SILC 2011, the proportion of missing keys for women was higher than for men among all age groups (by about 1 percentage point). As a consequence, using register data results in an under-reporting of income data for those groups. Most households with a reported household income of zero Euros receive an imputed income during the data editing process.^9^ However, for unlinked individuals in households with linkable people the under-reporting remains, resulting in household incomes that are too low on average.

Register data use affects both (1) the sources of the household income and (2) weighting. For the calculation of sample weights^10^ the wage tax income (number of recipients of income from employment and pensions) was used as a new marginal distribution to improve the consistency of the results compared to the income tax register (distribution of income) and to even out selective nonresponse bias for certain groups.

### Measuring Household Income and Poverty in EU-SILC

Total household income in EU-SILC is calculated as the sum of earned income, capital gains, pensions and public social transfers minus taxes and social security contributions plus the net flow of (paid/received) alimonies and other (paid/received) private transfers between households (UNECE 2011). Equivalised household income is defined as a household’s disposable income divided by the sum of consumption equivalents of that household using the modified OECD scale to reflect economies of scale: for each household the first adult receives a weight of 1, each additional adult gets a weight of 0.5 and each (additional) child under 14 years receives a weight of 0.3. The poverty indicator is based on the distribution of the equivalised household income. If it is below 60% of the median of its distribution, a household and all of its members are defined as poor. The poverty rate refers to the weighted percentage of people in poverty in a population.

Table 1 provides an overview of the data source for each income variable for 2010 before and after the revision. Income components highlighted in grey were derived from registers for the revision.^11^ Some variables are exclusively based on survey data due to the unavailability of register data sources or because of methodological reasons (e.g. the time lag for receiving final data for self-employment income PY050 is too long). When speaking of “register based household income” in this paper we refer to the revised household income compiled from this combination of register and survey data. However, we argue that this is sufficiently justified as the sum of all components from register data is rather high as percentage of the total amount (between 86.4% in 2011 and 87.5% in 2010).

**Table 1:** Calculation of the total household income in EU-SILC 2010

			Sum, billion €		% Persons with income >0^b^	
			Revision (register)	Before (survey)	Revision (register)	Before (survey)
+	PY010	Employee cash/near cash income^a^	75.727	73.984	57.9	55.7
+	PY050	Cash benefits/losses from self-employment	11.04	11.338		
+	PY090	Unemployment benefits^a^	3.291	2.771	12.6	9.7
+	PY100	Old-age benefits^a^	30.14	29.849	25.3	25.2
+	PY110	Survivor benefits^a^	0.568	0.571	1.3	1.1
+	PY120	Sickness benefits^a^	0.538	0.461	5.9	3.2
+	PY130	Disability benefits^a^	2.932	2.246	3.7	2.6
+	PY140	Education-related allowances	0.303	0.301		
+	PY080	Pension from individual private plans	0.144	0.138		
=		Sum of personal incomes	124.683	121.659		
+	HY040	Income from rental of a property or land	2.062	2.060		
+	HY050	Family/children-related allowances^a^	5.782	6.133	48.3	50.3
+	HY060	Social exclusion benefits not elsewhere classified	0.356	0.366		
+	HY070	Housing allowances	0.314	0.317		
+	HY080	Regular inter-household cash transfer received	1.227	1.224		
+	HY090	Interest, dividends, profit from capital investments in unincorporated business	1.740	1.741		
+	HY110	Income received by people aged under 16^a^	0.103	0.096	1.9	1.8
=		Sum of household incomes	11.583	11.937		
–	HY130	Regular inter-household cash transfer paid	1.564	1.573		
–	HY145	Repayments/receipts for tax adjustment^a^	−0.787	−0.620	5.4	6.4
**=**	HY020	Total disposable household income	135.488	132.643		

Statistics Austria, EU-SILC 2010. Weighted results. ^a^ Income components based on register data. ^b^ Rates for personal income components (“PY ….”) are calculated for persons aged >15 only. Values include imputations. Differences for non-register variables are due to weights based on register data. Full tables for all years are available in the online supplementary materials (Tables A1–A3)

Income from employment and family benefits are most common, with approximately 50% of the population receiving each of these types of income (Table 1).^12^ In general, the total weighted sum of household income captured by surveys is markedly lower than the register data. Furthermore, registers show a noticeably higher share of recipients of unemployment benefits, sickness benefits and employee income.

## Methods and Hypotheses

### Steps of the Analysis

The analysis comprises two main parts. The first part (Sect. 4.1) examines the factors that explain the observed differences in equivalised household income between register and survey data. Second, as the total household income is the source for calculating the poverty rate we then go on (Sect. 4.2) to investigate how the poverty rate and other income statistics are affected if the data source for income components is changed from surveys to registers.

**Fig. 1:**
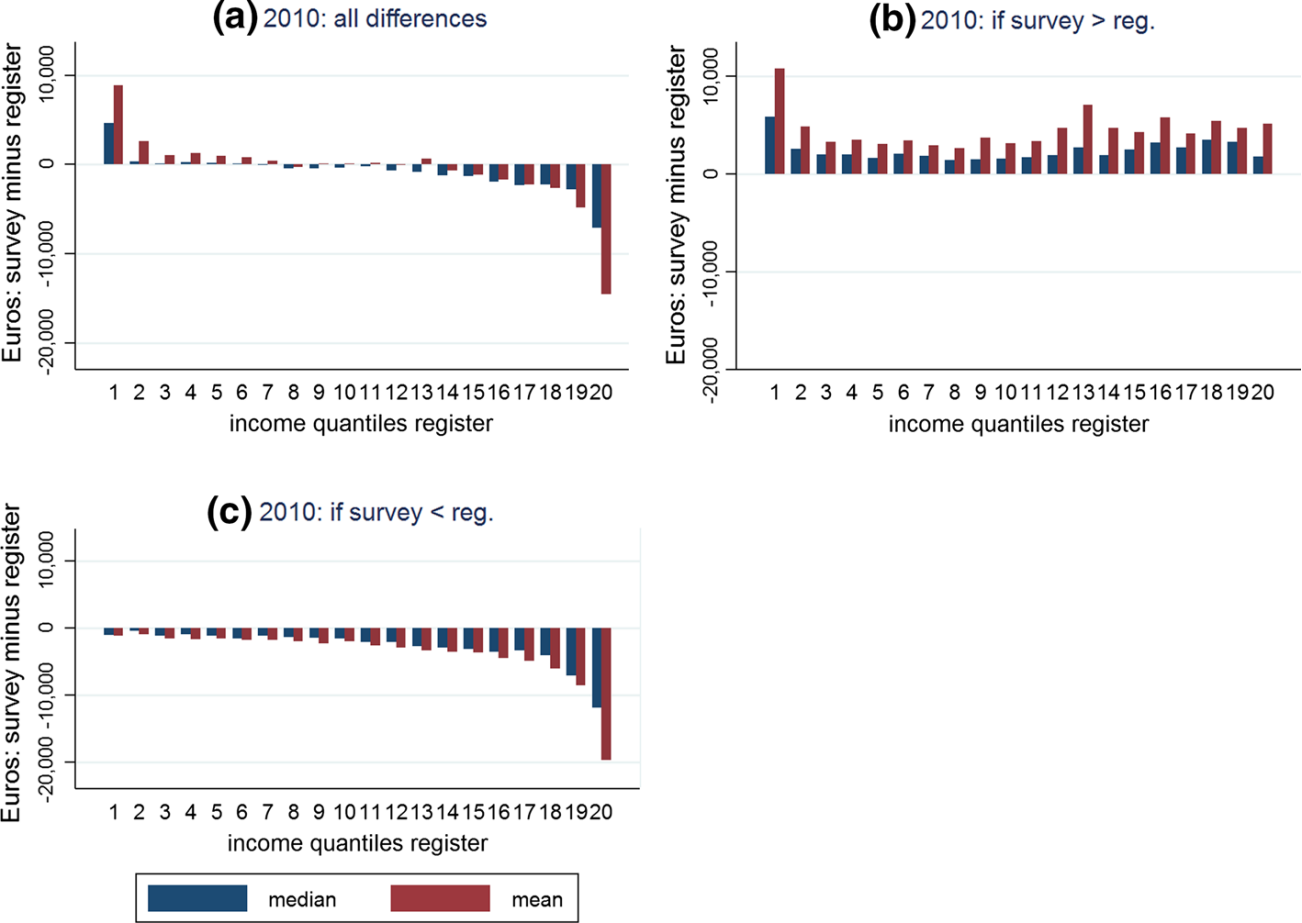
Weighted data. Median and mean of absolute deviation for equivalised household income for 20 quantiles derived from registers (2010). Persons are units of observation: **b** N = 8078, **c** N = 5913. Figure 4 in the “Appendix” contains the difference between data sources if equivalised household incomes are measured in logs. This procedure takes into account the current level of income and illustrates the relative deviation. A similar pattern then occurs, although the deviation in the upper tail of the distribution is markedly lower

For 56.5% of individuals (57.5% of all households) a negative difference (survey < register) for equivalised household income is found, whereas a positive deviation (register > survey) occurs for 42.8% of all individuals (41% of the households). Plotting means and medians of these observed differences along twenty income percentiles based on register data shows a clear tendency among higher-income groups to under-report/underestimate their income and vice versa for lower-income groups (Fig. 1). This pattern is more systematic for under-reporting than for over-reporting.^13^

Differences between survey data and register data are regarded as measurement error. We differentiate between two main explanations for measurement error (Bound et al. 2001): social desirability (aka interviewer bias; Groves et al. 2009) and cognitive errors (misunderstanding, retrieval and calculation problems). The multivariate analysis in Sect. 4.1 takes a closer look at this issue. By including (and thus controlling for) other possible determinants, we aim to investigate whether social desirability or cognitive error is more relevant as the mechanism behind the observed income differences between data sources.

The multivariate analysis uses three types of regression models. First, separate multinomial logit models (with alternative-invariant regressors) are estimated (Cameron and Trivedi 2005: 500). The dependent variable has three categories that mirror three groups of households for which odds-ratios, conditional on socio-economic characteristics^14^ and some survey mode aspects (see the next section), are estimated. It is coded “0” if the relative deviation^15^ of the survey income from the register income lies within the range of 0.95–1.05 (“almost perfect identity”^16^—reference category); “1” if above 1.05 (“over-reporting”) and “2” if below 0.95 (“under-reporting”).

Second, we also take a direct look at the magnitude of metric differences of equivalised household income between data sources and estimate OLS models with the same set of explanatory variables as in the previous step. This also makes it possible to alter the functional form of the regression between explanatory variables and income differences. We present specifications where both the income from registers (independent variable) and the difference between surveys and registers (dependent variable) are measured in absolute terms (Table 2; models 2, 5) and natural logs (Table 2; models 3, 6). The latter estimates coefficients that represent the effect of a 1% change in the independent variable on the corresponding  %-change of the dependent variable. Wald tests of the null hypothesis that the two alternatives (over-reporting vs. under-reporting) can be combined for all pairs of alternatives were rejected.^17^ Thus, positive and negative differences are modelled separately. Negative absolute differences (survey < register), however, have been converted to positive values to facilitate the interpretation of coefficients.

Third, the panel dimension of our dataset is exploited by applying panel regression models with household fixed effects and time fixed effects. Due to the focus on within-household change over time (as compared to between-household differences in the cross-section), panel regression models allow controlling for household characteristics that are not observed in the dataset but are constant over time (e.g. cognitive ability of all household members or past experience with surveys). This helps to increase the consistency of the estimates and decrease a (possible) selection bias for the independent variable(s) under investigation (Hsiao 2014).

Data for SILC Austria come from a representative probability sample that involves stratification (based on federal states and interviewer regions) and features households as the primary sampling unit (Glaser and Till 2010). We consider these complex sampling design features by applying STATA’s survey procedure to our regression estimation commands (Kreuter and Valliant 2007). This also implicates the use of sampling weights for all cross-sectional analyses (descriptive statistics and regression models). Households are the units of observation in all regression models. In the cross-sectional regression analysis, we focus on the year 2010 as this is the most recent year available with a high share of successful links of person-specific IDs to their register entries (97%) and where the data source differs for maximum number of income components.^18^ Full tables for all years are provided in the online supplementary materials.

### Modelling Household Income Differences: Explanatory Variables and Hypotheses

The main focus of the regression models is on the effect of equivalised household income taken from registers on over-/under-reporting. Based on the social desirability argument, it is expected that households with low incomes tend to report a higher income than they actually have. On the other hand, households with a higher income are expected to make themselves “poorer” in the interview situation. In the multivariate analysis we aim to control for variables that are correlated with income and the observed measurement error. This allows to adjust the observed effect of income on measurement error (Fig. 1) for those dimensions that are related to cognitive error (e.g. number of income components, job changes) or other interview effects (e.g. no. of years in the panel). If there remains a significant effect of income on over-reporting/under-reporting, this could ceteris paribus be interpreted as evidence for social desirability bias related to the level of income. Furthermore, we test whether effects that are found in the literature for mode variables but for different samples or different types of income (Sect. 1.1) are also present in the SILC data.

The remaining explanatory variables in the models can be differentiated into four groups. The first group is related to the structure of the household income. It is expected that households with a lower number of different income sources (see Table 1 for all components) should have a less complex income situation and thus be less likely to have measurement error (see Sect. 1.1). Moreover, we assume an underlying social norm that makes poverty undesirable (Bosma et al. 2015; Sutton et al. 2014; Walker et al. 2013) and thus hypothesize that a lower level of satisfaction with household income increases the likelihood of over-reporting. As different income components represent different proportions of the total household income (Table 1) and also different magnitudes of measurement error,^19^ we also include a categorical variable which captures the income source with the highest share of total household income.

The second group of variables is related to employment status. Main employment status refers to the selected respondent for household variables (according to the interview guidelines in SILC). Changes in the main employment status during the income reference period are aggregated over all household members. In addition to the number of different income sources, these two variables can serve as a proxy for how much fluctuation occurs in yearly income streams which could make recall problems more probable. Thus, it is expected that retirees and people who mainly do housework have the lowest likelihood of misreporting. Similarly, households with a higher number of changes in labor status are expected to be more likely to have measurement error. The direction of effects for these variables is assumed to be the same, regardless of whether they are positive or negative deviations.

The third group of explanatory factors contains information on the interview situation: the total number of proxy interviews, a binary indicator which indicates whether the household was surveyed with CATI (rather than CAPI), the interview month and how often the household has already participated in SILC. The discussion of existing studies (Sect. 1.1) has shown that the evidence for the effects of proxy interviews on income measurement error is mixed and may depend on whether (lower) social desirability or recall problems are the main driver behind the effect. We thus do not assume any ex-ante hypothesis for proxy interviews. Based on the literature review, it is also ambivalent whether there will be less income measurement error in our data set with an increasing number of panels rounds a household participated. However, we expect that a longer distance between the interview month and the last income reference year is generally associated with a higher likelihood of reporting errors due to recall problems. Concerning mode effects, the literature has demonstrated that respondents to CATI are more likely to present socially desirable answers and that this can lead to a downward bias concerning income inequality (Sect. 1.1). Furthermore, CATI does not allow the use of visual aids may leave the respondent less time to check income records resulting in an enhanced likelihood of cognitive error. Thus, CATI is expected to increase measurement error in general and to be associated with both more over- and under-reporting than CAPI.

The fourth group of explanatory factors comprises socio-demographic characteristics. These mainly serve as control variables for income. Thus, we do not make any assumptions on their effects. Furthermore, consistent evidence for some socio-demographic variables (e.g. sex, children in the household) is lacking (Bound et al. 2001). Age and sex refer to the response person for household variables in SILC. Education measures the highest completed level of education in the household. Health is measured as the household’s median of an ordinal variable capturing the self-assessed health of all household members over the age of 15. Household structure and population size at the place of residence are measured directly at the household level.

## Results

### Explaining Household Income Differences

The primary focus of this section is on the effects of income, income-related variables (income satisfaction, income structure, employment status variables) and the interview context (e.g. mode). We do not comment in detail on the outcomes for the remaining socio-demographic variables as they mainly serve as controls in the models (see Sect. 3.2).

#### Negative Deviations (Under-Reporting)

From Table 2 it is evident that, even after controlling for a variety of other variables, both the likelihood of under-reporting (compared to a close identity of incomes) and the magnitude of under-reporting significantly increase with rising register income. This outcome is stable for all four years. For instance, a one percent increase in equivalised household income raises the odds of reporting a lower income than actually found in administrative records by a factor of 3.667 (column 4). Every additional available Euro increases the difference between data sources in this group by approximately 22 cents (column 5). Similarly, a one percent increase in personal income raises this difference by almost 2 percent (column 6).

**Table 2:** Results from cross-sectional regression models (2010)

	Over-reporting (survey > register)			Under-reporting (survey < register)		
	(1) Mlogit	(2) OLS	(3) OLS (dep. var in logs)	(4) Mlogit	(5) OLS	(6) OLS (dep. var in logs)
Ln(Epinc)	0.244***		−0.489***	3.654***		1.983***
Epinc		−0.299***			0.223***	
Epinc squared		0.00000204***			0.00000194***	
Satisfaction with household income (median)^a^	1.228***	981.1***	0.220***	0.830***	−942.1***	−0.156***
Main income: employed	1.340	2894.4***	0.672*	1.112	853.2	−0.258
Main income: self-employment	0.790	2217.6*	0.591	0.239***	−1966.1	−0.823***
Main income: social transfers	1.049	2786.0***	0.531	1.162	738.7	−0.0332
Main income: old-age benefits	Ref. cat.	Ref. cat.	Ref. cat.	Ref. cat.	Ref. cat.	Ref. cat.
Main income: other private	0.984	9869.8***	0.727*	0.0892***	−8900.5**	−1.801***
Total no. of different income components	1.034	−377.9*	−0.0345	1.026	−292.7*	−0.0149
Activity status^b^: full time work	Ref. cat.	Ref. cat.	Ref. cat.	Ref. cat.	Ref. cat.	Ref. cat.
Activity status: part time work	0.845	−742.7	−0.0647	1.190	381.4	0.0129
Activity status: unemployed	0.682	−3039.4***	−0.392*	1.817**	1133.3**	0.331*
Activity status: retired	0.849	−1076.0	−0.0826	0.949	137.2	−0.174
Activity status: student, other	0.462**	−5359.2***	−0.426*	1.383	2218.5**	0.569**
Activity status: housework	0.778	−1748.5*	−0.234	1.610**	1488.1**	0.274*
No. hh members >15 with >1 employment	1.258	−159.0	0.0395	0.809	−1745.5***	−0.154
Employ. status changes: none	Ref. cat.	Ref. cat.	Ref. cat.	Ref. cat.	Ref. cat.	Ref. cat.
Employ. status changes: 1	1.464***	6.807	0.221**	1.383**	727.4*	0.147*
Employ. status changes: >1	1.843*	−174.2	0.462**	1.794*	817.6	0.295*
Age	0.968*	45.93**	0.00202	1.001	−20.04	−0.0169
Age squared	1.000*			1.000		0.000179*
Male	Ref. cat.	Ref. cat.	Ref. cat.	Ref. cat.	Ref. cat.	Ref. cat.
Female	0.992	85.31	0.0234	1.056	−7.855	0.0249
Household sickness (median)^c^	0.978	−80.02	0.00324	1.062	68.11	0.0323
Education: basic	1	0	0	1	0	0
Education: middle	1.415**	1104.7**	0.338**	0.874	−643.3	−0.119
Education: high	1.419*	1750.6***	0.437***	0.707*	−1884.3***	−0.346***
Education: specialized	1.755***	4251.6***	0.683***	0.805	−2871.6***	−0.275**
Retired household	0.678	82.35	0.156	0.921	1391.5	−0.281
Single HH, not retired	0.652**	−1891.1*	−0.312**	1.026	1485.8**	0.195**
MPH, no children	Ref. cat.	Ref. cat.	Ref. cat.	Ref. cat.	Ref. cat.	Ref. cat.
Single parent	0.393***	−2706.5**	−0.740***	0.803	1562.5*	0.0734
MPH, children	0.627***	−2138.6**	−0.475***	1.066	1634.3***	0.196**
Region: Vienna (capital, >1,000,000 inh.)	Ref. cat.	Ref. cat.	Ref. cat.	Ref. cat.	Ref. cat.	Ref. cat.
>100,000 inhabitants	1.113	−491.2	−0.0374	1.171	122.8	0.138
>10,000 inhabitants	1.011	−354.4	−0.128	1.205	−387.5	0.0374
≤10,000 inhabitants	1.045	−1003.0*	−0.139	1.129	187.3	0.159**
CAPI	Ref. cat.	Ref. cat.	Ref. cat.	Ref. cat.	Ref. cat.	Ref. cat.
CATI	1.046	482.2	0.142	0.936	−325.9	−0.0562
Sum of proxy interviews in hh	1.199*	12.81	0.0871	1.159*	139.6	0.0795
Interview month	1.030	−129.9	0.00267	1.036	164.2*	0.0216
SILC round 1	Ref. cat.	Ref. cat.	Ref. cat.	Ref. cat.	Ref. cat.	Ref. cat.
SILC round 2	0.999	−637.2	−0.0895	1.002	−477.2	−0.111
SILC round 3	0.896	−2255.7***	−0.357***	1.008	−36.69	−0.0338
SILC round 4	0.858	−1597.9***	−0.204	1.079	−219.8	−0.111
Constant		4592.0*	10.83***		1231.5	−11.25***
R^2^ (OLS models)		0.166	0.146		0.689	0.345
N (Households)	6074	2448	2448	6074	3546	3546

* *p* < 0.05, ** *p* < 0.01, *** *p* < 0.001. ^a^ scale from 1 to 6; 1 = very unhappy, 6 = very happy. ^b^ Self-reported labor status in income reference period for 2010 for more than 6 months. ^c^ scale from 1 to 5; 1 = very good 5 = very bad

*Multinomial logit regression models* using sampling weights: Coefficients of the model are estimated at once. Sample size in col. (1) and (4) refers to the sum over all 3 categories of the dependent variable. Coefficients show odds ratios. Odds For the dependent variable, the reference category refers to households with a difference between equivalised household incomes that lies within the range of ±5%. Standard errors (not displayed) account for complex stratified survey design. Pseudo R^2^ measures are not available for Maximum Likelihood estimation as the assumption of observations being independent and identically distributed (*iid*) is not fulfilled. MPH: Multiple person household. *Epinc* was used in log form after comparing the Akaike and Bayesian Information Criteria

*OLS regression models* using sampling weights: Dependent variable is *epincdelta* (quest. minus register). Standard errors (not displayed) account for complex survey design (strata = federal states). *Age squared* and *epinc squared* were only included in a model if the Wald test was significant

In the multinomial logit models (column 4), other statistically significant effects are found for households with the main income source coming from self-employment or private sources (as compared with those receiving old-age benefits). Increasing satisfaction with the household income lowers the odds for under-reporting compared to approximate equivalence between data sources. If the main activity status of the person answering the household questionnaire is housework, this raises the odds of under-reporting. A change in the labor status of the household also increases the odds of under-reporting, as does being unemployed.

The evidence for variables related to the interview context is mixed. A higher number of proxy interviews increases the odds of under-reporting, whereas the effects of CATI are statistically not significant.

The results of the OLS models (column 6) for metric differences resemble the outcomes of the multinomial logit models for the most part. The higher the number of different income components in the household, the lower the magnitude of under-reporting. This outcome may also suggest that using a detailed collection of income components to calculate the total household income (vs. a single question) does not affect data accuracy very much. The higher the satisfaction with the household income is, the lower the magnitude of under-reporting. The same applies if the main income source of the household is either self-employment or private resources (in comparison with old-age benefits as reference category). Housework or being in education as the main activity status of the respondent person for the household questionnaire and changes in labor status are associated with higher income differences. Contrary to expectations, the number of household members with more than one employment activity in the survey year has a negative effect on under-reporting. Households where the response person was unemployed for most of the income reference period have a significantly higher amount of under-reporting than households with the response person working full-time. Finally, when looking at variables closely related to the interview procedure itself, we do not find any relationship of the dependent variable with CATI. In contrast to the multinomial logit, the effects of the number of proxy interviews are not significant anymore.

#### Positive Deviations (Over-Reporting)

Both the likelihood of over-reporting (compared to a close identity of incomes) and the magnitude of over-reporting significantly decrease with rising income. For instance, a one percent increase in equivalised household income lowers the odds of reporting a higher income than actually found in administrative records by a factor of 0.244 (Table 2, column 1). A one percent increase in personal income lowers the difference by roughly 0.5 percent (column 3). In absolute terms (€), the relationship between income from registers and the magnitude of over-reporting is negative and marginally non-linear, i.e. the effect size slightly decreases with rising income (column 2).

In contrast to under-reporting, various significant effects are also found for other income-related variables. A one unit increase in satisfaction with household income is generally associated with a small but statistically significant increase in the odds of over-reporting (column 1) and a significant increase in the magnitude of over-reporting (columns 2, 3). If the main income source of the household is private resources (as compared with old-age benefits as the reference category) this raises the difference between data sources substantially (9869.80 €). Smaller significant positive effects are also found if income from employment or social transfers is the main income source. In contrast, to what was expected in Sect. 3.1, a rising number of different income components in the household decreases the absolute difference in the case of over-reporting.

Similar to the models for under-reporting, changes in household members’ labor status during the income reference period slightly increase the odds of over-reporting. Moreover, households where the response person was unemployed or in education most of the time during the income reference period, have a significantly lower amount of over-reporting than households with the response person working full-time (column 2, 3).

Variables related to the interview context do not seem to strongly influence the outcome variable. A statistically significant but rather weak positive effect on the odds of over-reporting was only found for the sum of proxy interviews in the household (column 1 & 4). CATI is not significant.

Taking together all model results for over-reporting and under-reporting three main conclusions can be drawn. First, income effects are as expected and also mirror the descriptive analysis: the magnitude of under-reporting rises with increasing income, whereas an opposite correlation can be found for over-reporting. However, the latter statistical relation is weaker in magnitude when compared with under-reporting. Second, for the OLS models (col. 2, 3, 5, 6) a generally higher model fit is observed for under-reporting. Third, variables related to the interview context play only a very modest role in explaining the dependent variables.

In a final step, we exploit the panel dimension of our dataset. 28.1% of all households in the unbalanced panel sample (at least 1 participation 2008–2011) have a mix of both positive and negative deviations from their equivalised household income as derived from administrative records. 28.5% have only positive deviations and 41.3% have only negative deviations. 2.1% have complete conformity for their equivalised household income from both data sources over their observation period. All panel regression models are estimated at the household level and control for time-constant unobservable household characteristics. The primary focus is on the effects of income and time (‘learning effect’; i.e. whether the difference between survey data and administrative data on average decreases over time). Due to space limitations we cannot comment in detail on the outcomes for other model variables. The panel models (Table 6, “Appendix”) provide additional evidence for the U-shaped relationship between equivalised household income and both the absolute and the logarithmic differences between survey and register data. Furthermore, we now see evidence for a learning effect, which was less pronounced in the cross-sectional analysis. There are fewer significant effects for other model variables, which may be partly due to the short observation period of only 4 years and the low with-in variation (resulting in higher standard errors for the estimates).

### How Changes in the Distribution of Equivalised Income and Its Components Affect the Poverty Rate

**Fig. 2:**
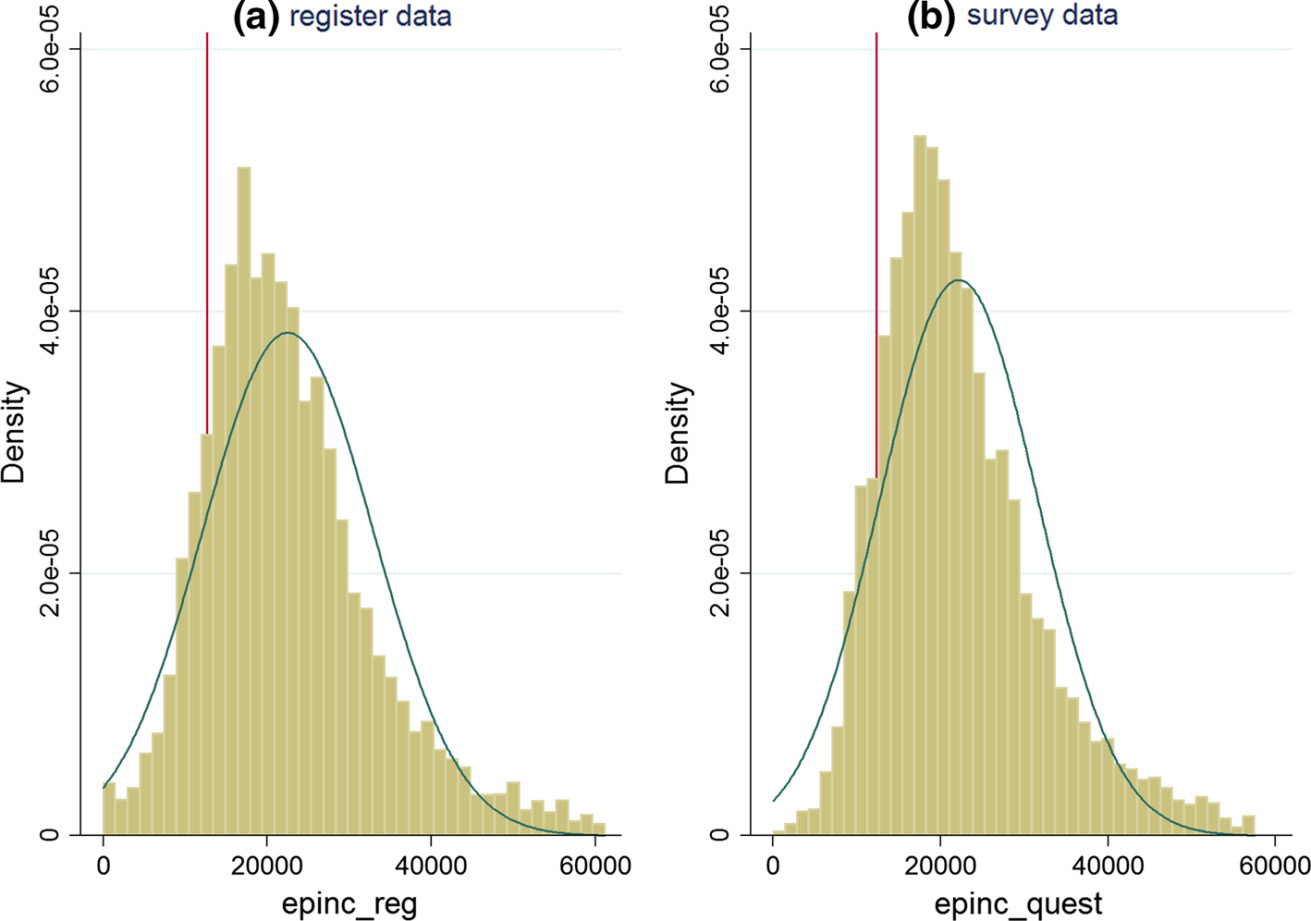
Weighted data. Histogram for the distribution of equivalised household income (2010). *Red line* = poverty threshold (€). Top 1% excluded for better readability. Persons are units of observation

Table 3 shows that the poverty rate is persistently underestimated in all four years if based on survey data. Except for 2011, there is also an increase in the poverty gap (distance of the median income of the poor to the poverty threshold as percentage of the threshold) of 3–4 percentage points. Furthermore (not shown in Table 2), longitudinal poverty (4 times poor out of 4 times 2008–2011) also increases from 4.8% to 7.1%. As the poverty rate is calculated based on total household income, changes in its distribution translate directly into changes in the poverty rate. The Gini coefficient and the relation of the income at the 90^th^ percentile to the 10^th^ percentile indicate that total household income inequality increases if registers are used. Both the median and mean incomes are slightly higher. Except for 2008, the total income found in registers also varies more, as indicated by the standard deviation. Furthermore, Fig. 2 shows that there is more probability mass at the lower end of the distribution based on register data. Taken together, this evidence indicates that the rise in the aggregate poverty rate is in part explained by a higher number of households with a very low income than in the survey data.

**Table 3:** Poverty indicators and the distribution of equivalised household income for different data sources

	2008		2009		2010		2011	
	Survey	Reg.	Survey	Reg.	Survey	Reg.	Survey	Reg.
*Poverty indicators*								
% At risk of poverty	12.4	15.2	12.0	14.5	12.1	14.7	12.6	14.5
Poverty threshold, €	11,406	11,648	11,931	12,281	12,371	12,635	12,791	12,878
Poverty gap, %	15.3	19.9	16.9	19.2	17.2	21.8	19	19.1
*Equivalised income*								
Gini	0.26	0.28	0.26	0.27	0.26	0.28	0.26	0.27
p90p10	3.146	3.383	3.057	3.268	3.185	3.457	3.087	3.330
Mean	21,381	21,679	22,098	22,750	23,158	23,596	23,642	23,922
Median	19,011	19,413	19,886	20,469	20,618	21,058	21,319	21,463
SD	13,739	12,783	11,786	13,501	12,736	14,654	13,525	13,561

Weighted data. Poverty Gap = (median income of the poor − poverty threshold)/poverty threshold × 100. Persons are units of observation

**Fig. 3:**
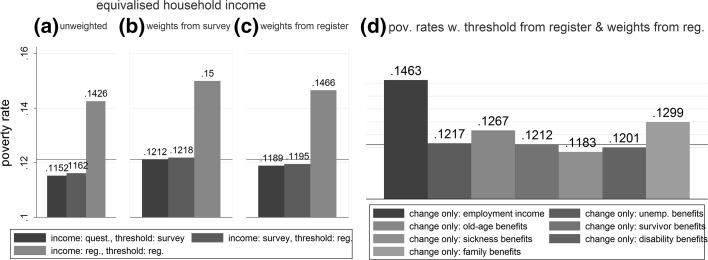
Poverty rates (1 = 100%) for 2010 based on income components and/or weights from different data sources. The *red reference line* represents the poverty rate if both income data and the poverty threshold are derived from surveys (12.2%). Persons are units of observations

By definition, household income, the poverty threshold and the sample weights determine the poverty rate in arithmetical terms. The left panel of Fig. 3 illustrates the effect of changing the data source from surveys to register for either only the poverty threshold (red bar) or both household income and the threshold (green bar). This is compared to a baseline where both income and the calculation of the threshold is based on survey data (blue bar). The red reference line marks the “old” poverty rate calculated with both sample weights and income data from surveys. If nothing else except the source for the sample weights is changed (compare *c* to *b*), the poverty rate decreases by less than one percentage point. A similar conclusion but with a different direction of change can be drawn for the poverty threshold (compare blue to red bar within a, b, c). In contrast, irrespective of sampling weights, altering the data source for household income leads to marked upsurges in the poverty rate of 2.5–3 percentage points (compare blue to green within a, b, c).

The right panel (d) of Fig. 3 simulates the effects of a change from survey data to register only for single income components. Such a change leads to a different household income and subsequently alters the overall distribution of all household incomes including the median and poverty threshold in a given year. Each bar thus represents the poverty share in the population based on a corresponding new poverty threshold. Changing the source for income from employment clearly has the greatest effect on the poverty rate. Given that 58% receive this type of income (Table 1) this is a predictable outcome. Moreover, family/children-related allowances and old-age benefits also noticeably increase the aggregate poverty rate, whereas sickness benefits and disability benefits decrease the poverty rate if derived from registers.

As changes in the poverty rate are strongly driven by differences in employment income, the question arises whether the differences measured between data sources themselves are particularly sizeable with regard to this income component. Rows (a) and (b) of Table 4 contain descriptive statistics on the distribution of differences among particular income components after the change from survey to register data. Differences are grouped with respect to poverty status changes due to the data switch. Those who enter poverty clearly over-report their employment income with a higher magnitude (≈90%) than those who exit poverty (≈−25%), both in absolute and relative terms. Moreover, we find relative differences of similar magnitude for old-age benefits. Those who are newly classified as poor based on register data also have a markedly higher share of family benefits as part of total household income (row c). Furthermore, the median relative distance of the new equivalised income to the new poverty threshold (not shown in Table 4) is 17.5% for households switching from poor to non-poor and −18.8% for households switching from non-poor to poor, whereas the median relative distance of the old equivalised income to the new threshold amounts to −13.6% for the exits and 25.3% for the entries.

**Table 4:** Effects of using register data on income statistics for those whose poverty status changes thereof, 2010

	Indicator	Poverty status	Equivalised household income	Employment income	Unemployment income	Old age benefits	Family benefits
(a)	Survey minus register: Median of absolute difference (€)	Exit	−4151.7	−2819.50	−310.5	−2890.9	0.0
		Enter	6784.5	5301.10	−175	4478.3	71.5
(b)	Survey minus register: median of relative difference (%)	Exit	−29.1	−24.8	−9.0	−21.6	0.0
		Enter	76.6	89.9	−9.9	48.1	1.0
(c)	Median (%): sum of this component in HH as % of household income (register)	Exit	n.a.	49.8	20.3	68.6	16.1
		Enter	n.a.	53.4	19.0	93.4	30.3

(a), (b) observations with zero income in both survey and register are excluded. n.a., not applicable; HH, household. Persons are units of observations

### Robustness Checks

Additional checks and analysis beyond our main models deal with (1) the robustness of the cross-sectional models’ results for the other three available years, (2) several specification tests concerning the functional form and (3) a comparison of register data against a single survey question on total household income available in the questionnaire. Detailed results of all these estimations beyond the summary in this section are provided in the online supplementary materials.

Overall, repeating the estimation of the main models (Table 2) for the remaining three years 2008, 2009, 2011 again reveals that both the odds and magnitude of under-reporting generally rise with increasing income, whereas an opposite correlation can be found for over-reporting. However, the latter statistical relation is less robust over time (sometimes insignificant) and weaker in magnitude when compared with under-reporting: there are small negative effects for income which, however, are only statistically significant in 2010 and 2011. Using logarithmic values for the magnitude of measurement error (dependent variable) and income from register data (independent variable) instead of levels does not substantially change the statistical significance and direction of parameter estimates for the explanatory variables. However, the OLS models with logarithmic values of under-reporting and income yield lower fit statistics for all years except in 2011 as compared to the specifications in levels.

A series of specification tests were applied to validate the robustness of the main results reported in Table 2. First, Box-Cox transformations (dependent variable) were used to check whether the OLS regression model for the dependent variable is better in logs than in levels (Cameron and Trivedi (2005), chapter 8.5.2, *boxcox* command in STATA). Second, J-tests and Cox-Pesaran tests for non-nested OLS models were applied to choose between income on the right-hand side of the equation to be specified in logs or in levels (*nnest* command in STATA, see Greene (2000): 302–305). This procedure did not yield completely unambiguous results. However, for positive differences (survey > register), it generally indicated that models with the left-hand side income variables measured in levels and measured the right-hand side income variables measured in logs may suit the data better than using models with levels for both. We are also aware of potential problems in log–log models due to heteroscedasticity (Silva and Tenreyro 2006). Consequently, we also estimated Poisson regression models, which are suggested as one solution for this problem (Martinez-Zarzoso 2013; Santos Silva and Tenreyro 2011; Silva and Tenreyro 2006). In sum, substantial results of our study are robust to these tests and do not crucially depend on the functional form of the regression model.

Finally, some studies have shown that measurement error can be of different magnitude whether total household income is calculated based on a single survey question or aggregated based on multiple income questions. Using data for the UK, Hansen and Kneale (2013), find that households with more diverse sources of income, such as the self-employed, part-time employed and those in receipt of means-tested benefits, were more likely to report higher incomes when using multiple income questions compared to using a single question. A study on behalf of Eurostat (Dia et al. 2013) concluded that measurement of yearly income based on several components is more accurate and complete than current monthly income.

The Austrian SILC also contains a single question on current monthly household income.^20^ Thus, we checked whether the observed effects of our main models apply to a lesser or greater extent if register data are compared to this single question on current monthly household income. To construct the dependent variable, the difference between this variable (equivalised for household size) and 1/12 of the equivalised annual household income from register was calculated. For the construction of the dependent variable for the multinomial logit model, we used the same thresholds as four the model in Sect. 4. The median of the relative deviation [(survey minus register)/register*100] amounts to amounts to 25% as compared to 10% when measurement error is calculated as described in Sect. 3.1. Overall, the estimated effects of income on measurement error have the same direction and significance as the regression models in Sect. 4.1. A one percent increase in equivalised monthly household income derived from a single question lowers measurement error by roughly 0.7% in the case of over-reporting and increase measurement error by 1.6% in the case of under-reporting. Again, a mean-reverting relationship between income and measurement error is observed.

## Conclusion and Discussion

The aim of this paper is to investigate the consequences of substituting survey data with register data in Austria for income measurement at the household level and how this affects the poverty rate based on a threshold relative to the median income.

In the multivariate analysis, differences between the two data sources for the same observations were regressed on income variables, socio-demographic variables and variables related to the interview context. One the one hand, both the likelihood of under-reporting and the magnitude (metric differences) of under-reporting significantly increase with rising income. This outcome is also relatively stable for all of the four years. On the other hand, the likelihood and magnitude of over-reporting significantly decreases with rising income. Panel regression results reflect these outcomes and complete the picture of mean-reverting errors (differences) when measuring disposable household income. Furthermore, a generally higher model fit is observed for under-reporting.

A different question is whether these income effects found are mainly due to cognitive error or social desirability. Controlling for variables that are correlated with income and the observed measurement error allows to adjust the observed effect of income on measurement error for those dimensions that are related to cognitive error (e.g. number of income components, job changes) or other interview effects (e.g. no. of years in the panel). As there remains a significant effect of income on over-reporting/under-reporting in our models, this could ceteris paribus be interpreted as evidence that it is primarily social desirability bias related to the level of income that underlies the observed pattern. However, effect heterogeneity between income groups is also possible. For instance, even after controlling for the number of different income sources in the household, cognitive errors may become increasingly important as income rises. In consequence, this could render the interpretation of the effect size and effect direction more ambiguous for higher-income groups.

Besides the relationship between income and the measured difference, we also find evidence for a “learning effect”: differences between data sources for both under-reporting and over-reporting decrease with the number of panel waves a household has participated in. Whether this effect occurs because respondents feel less uncomfortable reporting their income over time or due to better preparation and knowledge of one’s income data over time, however, does not have a straightforward answer based on the available data. Among the other variables related to the interview context only the number of proxy interviews (weakly) increases the odds of under- and over-reporting.

Finally, the analysis reveals a quite significant increase in the cross-sectional poverty rates for 2008–2011 and the longitudinal poverty rate if register data are used, whereas central measures of equivalised household income remain rather unchanged. The income distribution becomes more uneven when using register data. At the lower tail of the distribution the median income increases, whereas the opposite is true for the upper tail of the income distribution. Using register data also results in a higher number of households with a very low income as compared to survey data. Overall, the observed changes in the poverty rate are mainly driven by differences in employment income rather than sampling weights and other income components. Solely changing the source for the sample weights has only a very moderate effect.

Further research endeavors could test the implications of these outcomes for poverty dynamics. For instance, under-estimation of the poverty headcount in the cross-section based on questionnaire data may lead to a higher rate of households’ mobility into and out of poverty that is higher than the actual rate. As a consequence, poverty may turn out to be more persistent based on register data. Moreover, it could be investigated if statistical relationships between material deprivation indicators available in SILC and income poverty are altered significantly if register data are used. Further research could also clarify if social desirability is more relevant for some income types than for others (e.g. social benefits vs. market income).

### Electronic supplementary material

Below is the link to the electronic supplementary material.

Supplementary material 1 (DOCX 829 kb)

## Figures and Tables

**Fig. 4:**
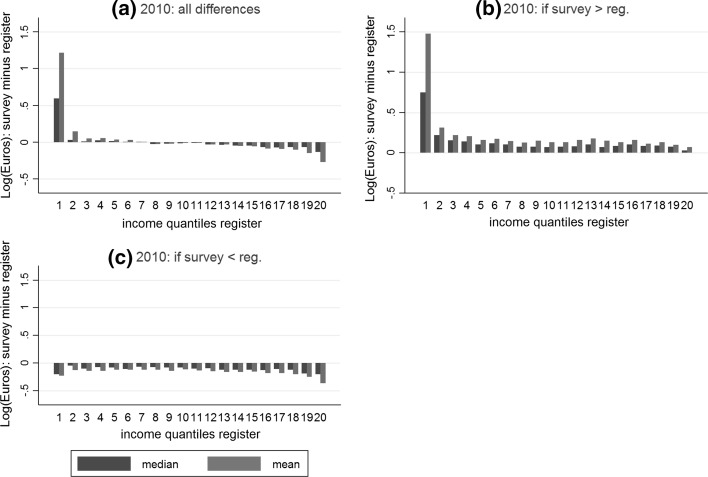
Median and mean of the difference between data sources if equivalised household incomes are measured in logs. Differences displayed for 20 quantiles of equivalised household income derived from registers (2010). Weighted data

## Appendix 1: Short Description of Register Data Sources for the Austrian SILC (cf. Statistics Austria 2014)

Based on the national regulation ‘*Einkommens*- *und Lebensbedingungen*-*Statistikverordnung* - *ELStV*’ the sector-specific personal identifier (bPK) allows an anonymized linking of survey and administrative data for the sampled individuals (§6). The following list contains a description of those national registers that were used for collecting income variables.

- *Dataset on HV*-*Qualifications* contains entries from Austria’s public social security register. This record does not include income information, but a number of variables concerning the social security status or changes in social security status for a given year, e.g. whether somebody received unemployment benefits, disability benefits etc (2008–2011).
- *Wage Tax Dataset* contains information on all taxable earnings; mainly employee earnings, pension income (retirement benefits), paid maternity leave (8 weeks before and after expected birth date) and sickness benefits. The data set contains information on gross income, paid social security contributions and payroll taxes paid. It thus allows the calculation of the respective net earnings. Moreover, the wage tax dataset also comprises care allowances, although they are not taxable in Austria. In sum, the wage tax dataset delivers roughly 75% of the total household income in EU-SILC (2008–2011).
- *Pension dataset* data on old-age benefits received from the public pension system. This dataset is also a source for income target variables other than PY100 (2008–2011).
- *Tax adjustment dataset* total repayments or receipts for tax adjustment in a given year for employee earnings (irrespective for how many years the tax was adjusted) (2008–2011).
- *Transfers dataset* data on unemployment benefits (on a daily basis); the dataset also contains the beginning and ending date of unemployment spells (=beginning and terminating of benefits) (2008–2011).
- *Dataset on family allowances* information on family/children-related allowances. A differentiation with respect to the number of children or other characteristics of the children (disabilities) is not possible. One total sum for each bPK-AS is provided (2008–2011).
- *Dataset for benefits to accident victims and surviving dependents* (2010 and 2011).

## Appendix 2

See Table 5.

**Table 5:** Equivalised household income (EPINC): differences between data sources

%	2008	2009	2010	2011
EPINC_quest = EPINC_reg	1.8	1.4	1.4	10.4^a^
[EPINC_quest/EPINC_reg] × 100 = in the range of ±5%	27.4	29.2	29.8	36
2 = <[EPINC_quest/EPINC_reg] > 1.05	28.7	26.4	25.4	20.1
0.5 = <[EPINC_quest/EPINC_reg] < 0.95	35.9	36.8	37.5	29.4
[EPINC_quest/EPINC_reg] > 2	4	3.9	3.9	2.5
[EPINC_quest/EPINC_reg < 0.5]	2.1	2.3	2	1.5
Total (%)	100	100	100	100
N (Households)	5707	5876	6188	6187

Weighted data. Households are units of observation

*EPINC_reg* equivalised household income from registers, *EPINC_quest* equivalised household income from questionnaire

^a^The higher overlap in 2011 is in part due to the fact that old age benefits were already drawn from registers in the primal data collection round

## Appendix 3

See Fig. 4.

## Appendix 4

See Table 6.

**Table 6:** Results from panel regression models (four rounds 2008–2011) with household fixed effects

Dep. var. is epinc_survey_ minus epinc_register_	Levels		Logs	
	Unbalanced	Balanced	Unbalanced	Balanced
Equivalised income: Lowest 5%	2789.6***	3455.5***	0.623***	0.645*
10% percentile	362.2	1001.4	−0.137	−0.0259
15% percentile	−392.9	474.2	−0.153	−0.316
20% percentile	−141.6	84.01	−0.102	−0.212
25% percentile	−107.3	741.8	−0.169	0.0488
30% percentile	28.09	290.4	−0.124	−0.0205
35% percentile	−56.75	760.2	−0.125	0.147
40% percentile	−83.13	1141.7*	−0.141	0.204
45% percentile	−52.16	545.3	0.0269	0.223
50% percentile	Ref. cat.	Ref. cat.	Ref. cat.	Ref. cat
55% percentile	435.8	1139.8**	0.0802	0.153
60% percentile	643.6*	318.9	0.0977	0.203
65% percentile	915.2**	1171.1	0.249**	0.430*
70% percentile	1002.7**	1007.9	0.151	0.182
75% percentile	1341.0***	1689.1*	0.171	0.440*
80% percentile	1719.5***	2128.5*	0.367***	0.466*
85% percentile	2577.3***	2921.1**	0.495***	0.709***
90% percentile	4314.4***	4498.0**	0.705***	0.916***
95% percentile	5704.8***	5858.0***	0.832***	1.130***
Top 5%	13600.1***	11,561.7***	1.340***	1.533***
HH Satisfaction w. household income (median)^a^	72.55	40.31	0.0276	0.00866
Main income: employed	1376.6	763.1	−0.490**	−0.467
Main income: self-employment	−3354.6**	−1941.5	−0.814***	−0.983*
Main income: social transfers	289.1	1181.1	−0.850***	−0.617
Main income: old-age benefits	Ref. cat.	Ref. cat.	Ref. cat.	Ref. cat
Main income: other	−1082.7	−2086.8	−0.496*	−0.602
Total no. of different income components	−87.99	−137.0	−0.0620***	−0.0412
Activity status^b^: full time	Ref. cat.	Ref. cat.	Ref. cat.	Ref. cat
Activity status: part time	−70.30	−1076.9	−0.0134	−0.135
Activity status: unemployed	−548.0	−1205.7	−0.0246	−0.0890
Activity status: retired	329.0	−171.1	−0.165	−0.223
Activity status: student, school, other	215.6	410.0	0.148	0.256
Activity status: housework	−428.5	−230.0	−0.0388	0.0546
No. hh members >15 with >1 employment	47.44	−541.4	0.0712	0.0305
0 employment status changes	Ref. cat.	Ref. cat.	Ref. cat.	Ref. cat
1 employment status change	319.6	426.5	0.183***	0.182*
>1 employment status changes	693.6	1223.7	0.210*	−0.0368
Household sickness (median)^c^	−141.7	−90.97	0.0196	0.00281
Education: basic	Ref. cat.	Ref. cat.	Ref. cat.	Ref. cat
Education: middle	168.0	362.7	0.0843	0.116
Education: high	−12.68	327.2	0.0721	0.0598
Education: specialized	−688.0	1045.0	−0.0125	0.0766
Retired household	566.4	933.1	−0.00615	−0.104
Single household, not retired	−213.4	−911.5	−0.733***	−0.838***
Multiple person household no children	Ref. cat.	Ref. cat.	Ref. cat.	Ref. cat
Single parent household	−397.7	564.6	−0.427**	−0.276
Multiple person household w. children	−309.7	−412.5	−0.00490	−0.204
Region: Vienna (capital city, >1,000,000 inh.)	Ref. cat.	Ref. cat.	Ref. cat.	Ref. cat
>100,000 inhab.	6506.7	407.5	0.217	−0.179
>10,000 inhab.	5444.5	1255.9	−0.0349	−0.604
≤10,000 inhab.	6534.1	1520.7	0.0496	0.394
CAPI	Ref. cat.	Ref. cat.	Ref. cat.	Ref. cat
CATI	−48.02	291.4	0.0929*	−0.0101
Sum of proxy interviews in household	−88.62	−92.91	−0.0344	0.0282
Interview month	82.35*	40.14	0.0406***	−0.0198
SILC round 1	Ref. cat.	Ref. cat.	Ref. cat.	Ref. cat
SILC round 2	−180.7	77.68	−0.228***	0.0657
SILC round 3	−689.4***	−199.1	−0.434***	0.0311
SILC round 4	−450.7*	−918.2***	−0.473***	−0.611***
N (Households)	23,585	4395	22,746	4228

* *p* < 0.05; ** *p* < 0.01; *** *p* < 0.001. OLS regression models with fixed effects for households. Unweighted estimates. Socio-demographic variables with low within-variation were not included in the model. Estimates for unbalanced panel (2008–2011) and 4 wave balanced panel sample (2008–2011). ^a^ scale from 1 to 6; 1 = very unhappy, 6 = very happy. ^b^ Self-reported labor status in income reference period for 2010 for more than 6 months. ^c^ Scale from 1 to 5; 1 = very good 5 = very bad. HH… Household

## Footnotes

1 The terms “register data” is used in this paper for income data that come from administrative sources, i.e. their intended purpose was not the statistical use but some other general or specific demand in the administration of a public body. We refer to it synonymously with “administrative data”. They are thus to be distinguished from data derived from a survey based on questionnaires (“survey data” SD).

2 For more information on the survey see the Eurostat Website under the link http://ec.europa.eu/eurostat/web/income-and-living-conditions/overview (accessed on Sep. 12, 2016).

3 Namely: Norway, Island, Turkey, Switzerland, Former Yugoslav Republic of Macedonia and Serbia.

4 Summary of the 2012 Workshop on Registers in the context of EU-SILC presented at the EU-SILC Task Force Legal Revision meeting in February 2013.

5 Cf. the work done in CHINTEX on data of the ECHP, e.g. Nordberg et al. (2001).

6 Verordnung des Bundesministers für Arbeit, Soziales und Konsumentenschutz über die Statistik der Einkommen und Lebensbedingungen, BGBl. II Nr. 277/2010.

7 Instead, persons and households participating in EU-SILC in Austria do so on a completely voluntary basis and are thoroughly informed about data linkage.

8 The reason here is that the inhabitants of Vienna more often have a non-Austrian background (country of birth or citizenship) and are therefore more likely to have a missing identifier.

9 This applies to households in which it is not possible to link any person to register data as well as for persons without any employee income, pension income or unemployment income based on the monthly main activity status.

10 General aspects of weighting are described in the Quality Reports publicly available on the CIRCA platform https://circabc.europa.eu/faces/jsp/extension/wai/navigation/container.jsp (09/05/2016).

11 However, due to register data limitations for some income components like family/children-related allowances (HY050), both survey and register data had to be used.

12 After changing the data source for some individuals, a particular component is no longer observed (=0), whereas for others an entry >0 can now be found in the registers. Thus, Table 1 represents the net outcome of these ‘inflows’ and ‘outflows’.

13 These patterns are very similar in all years observed (see Figure A1–A4 in the online supplementary materials).

14 Table A4 in the online supplementary materials provides an overview of the share of over- and under-reporting individuals from different social groups for the year 2010.

15 Relative deviation = (income from survey − income from register)/(income from register).

16 This bandwidth was chosen as there are hardly any households for which a perfectly identical equivalised income between the two data sources is observed (Table 5 in the “Appendix”).

17 Tables are available from the authors upon request.

18 In EU-SILC 2011, pension incomes were already derived from registers before the back-calculation.

19 In 2010, the median of the relative deviation [(survey minus register)/register × 100] amounts to 16% (employee income), 52% (unemployment benefits), 9% (old-age benefits).

20 Respondents are asked to think of a typical month to address the problem of seasonal effects when asking for monthly income.
