# Supplementary material for: Differences Between Household Income from Surveys and Registers and How These Affect the Poverty Headcount: Evidence from the Austrian SILC
Source: Soc Indic Res. 2017 Jun 12;138(2):575–603. doi: 10.1007/s11205-017-1672-7 (PMC6015103; doi:10.1007/s11205-017-1672-7)
Supplement: Supplementary file 1 — Supplementary material 1 (DOCX 829 kb) [file 11205_2017_1672_MOESM1_ESM.docx]

**Differences between household income from surveys and registers and how these affect the poverty headcount. Evidence from the Austrian SILC**

Online Supplementary Materials

**Article Section 2 *Data***

Table A 1 Calculation of the total household income in EU-SILC 2008 – 2011 2

Table A 2 Percentage of individuals with a particular income component >0 2

Table A 3 Total sum of income components for different data sources, bn. € 3

**Article Section 3 *Methods and Hypotheses***

Table A 4 Over-reporting and Underreporting among different groups 5

Figure A 1 Median and mean of absolute deviation for equivalised household income for 20 quantiles 6

Figure A 2 Median and mean of absolute differences > 0 for equivalised household income for 20 income quantiles 7

Figure A 3 Median and mean of absolute differences < 0 for equivalised household income for 20 income quantiles 8

Figure A 4 Median and mean of log differences for equivalised household income for 20 income quantiles 9

**Article Section 5 *Robustness Checks***

*A) Repeating the main analysis for the years 2008, 2009, 2011*

[Table A 5 Results from multinomial regression models – odds ratios, EU-SILC 2008 – 2011 10](#_Toc479596456)

[Table A 6: Results from regression models – OLS, dependent variable is epincdelta (survey minus register) 12](#_Toc479596457)

[Table A 7: OLS regression models. Dependent variable measured in logs, all years 2008-2011 14](#_Toc479596458)

*B) Specifications with different functional forms*

[Table A 8: OLS models. Level-log specification for income difference (>0) and income on right hand side 16](#_Toc479596215)

[Table A 9: Poisson Regression Models 18](#_Toc479596216)

*C) Register data compared with a single question on income measurement*

Figure A 5 Median and mean of absolute (left) and log(deviation) (right) for equivalised household income for 20 quantiles 20

Table A 10: Cross-sectional regression models (2010), dependent variable derived from single question 21

Table A 11: Results from panel regression models (4 rounds 2008 to 2011) with household fixed effects, dependent variable derived from single question 23

Table A 1: Calculation of the total household income in EU-SILC 2008 – 2011

|  |  |  | Sum, billion € | | | |
| --- | --- | --- | --- | --- | --- | --- |
|  |  |  | 2008 | 2009 | 2010 | 2011 |
| + | PY010 | Employee Cash or Near Cash Income | 69.918 | 73.585 | 75.727 | 74.221 |
| + | PY050 | Cash Benefits or Losses from Self-Employment | 10.181 | 11.162 | 11.04 | 12.056 |
| + | PY090 | Unemployment Benefits | 2.372 | 2.395 | 3.291 | 3.291 |
| + | PY100 | Old-Age Benefits | 26.153 | 27.88 | 30.14 | 28.617 |
| + | PY110 | Survivor Benefits | 0.389 | 0.531 | 0.568 | 3.163 |
| + | PY120 | Sickness Benefits | 0.535 | 0.563 | 0.538 | 0.715 |
| + | PY130 | Disability Benefits | 2.327 | 2.778 | 2.932 | 2.913 |
| + | PY140 | Education-Related Allowances | 0.283 | 0.305 | 0.303 | 0.328 |
| + | PY080 | Pension from Individual Private Plans | 0.214 | 0.133 | 0.144 | 0.249 |
| = |  | Sum of Personal Incomes | 112.372 | 119.333 | 124.683 | 125.553 |
| + | HY040 | Income from Rental of a Property or Land | 1.975 | 1.509 | 2.062 | 2.135 |
| + | HY050 | Family/Children-Related Allowances | 5.506 | 6.034 | 5.782 | 6.569 |
| + | HY060 | Social Exclusion Benefits not elsewhere classified | 0.249 | 0.231 | 0.356 | 0.34 |
| + | HY070 | Housing Allowances | 0.304 | 0.266 | 0.314 | 0.367 |
| + | HY080 | Regular Inter-Household Cash Transfer received | 1.233 | 1.154 | 1.227 | 1.411 |
| + | HY090 | Interest, Dividends, Profit from Capital Investments in Unincorporated Business | 1.862 | 1.660 | 1.740 | 1.904 |
| + | HY110 | Income received by People aged under 16 | 0.218 | 0.196 | 0.103 | 0.18 |
| = |  | Sum of Household Incomes | 11.347 | 11.051 | 11.583 | 12.908 |
| - | HY130 | Regular Inter-Household Cash Transfer paid | 1.363 | 1.410 | 1.564 | 1.549 |
| - | HY145 | Repayments/Receipts for Tax Adjustment | -0.788 | -0.842 | -0.787 | -0.881 |
| **=** | **HY120** | **Total Disposable Household Income** | **123.144** | **129.816** | **135.488** | **137.793** |

Note: Statistics Austria, EU-SILC 2008-2011. Weighted results. Shaded income components are based on register data.

Table A 2: Percentage of individuals with a particular income component >0

| % | Employment income^a^ | Unemployment benefits^1^ | Old-age benefits^1^ | Sickness benefits^1^ | Disability benefits^1^ | Family benefits |
| --- | --- | --- | --- | --- | --- | --- |
| 2008 Register | 56.6 | 10.2 | 24.1 | 5.2 | 3.4 | 49.1 |
| 2008 Survey | 53.8 | 8.3 | 24.9 | 3.1 | 2.8 | 51.9 |
| 2009 Register | 58.0 | 10.2 | 24.7 | 5.5 | 4.1 | 50.7 |
| 2009 Survey | 54.8 | 8.1 | 25.0 | 3.3 | 2.7 | 51.6 |
| 2010 Register | 57.9 | 12.6 | 25.3 | 5.9 | 3.7 | 48.3 |
| 2010 Survey | 55.7 | 9.7 | 25.2 | 3.2 | 2.6 | 50.3 |
| 2011 Register | 57.5 | 12.0 | n.a. | 6.3 | 4.4 | 52.0 |
| 2011 Survey | 55.8 | 9.2 | n.a. | 3.0 | 2.8 | 51.6 |

Note: Statistics Austria, EU-SILC 2008-2011. Weighted results. In 2011, old-age benefits were already drawn from registers in the primal data collection round. ^1^ Rates for these personal income components are calculated for persons aged > 15 only.

Table A 3: Total sum of income components for different data sources, bn. €

| bn. € | Employment income | Unemployment benefits | Old-age benefits | Sickness benefits | Disability benefits | Family benefits |
| --- | --- | --- | --- | --- | --- | --- |
| 2008 Register | 69.918 | 2.372 | 26.153 | 0.535 | 2.327 | 5.506 |
| 2008 Survey | 67.456 | 2.452 | 27.337 | 0.533 | 2.120 | 5.782 |
| 2009 Register | 73.585 | 2.395 | 27.880 | 0.563 | 2.778 | 6.034 |
| 2009 Survey | 69.952 | 2.302 | 28.667 | 0.541 | 2.220 | 6.325 |
| 2010 Register | 75.727 | 3.291 | 30.140 | 0.538 | 2.932 | 5.782 |
| 2010 Survey | 73.984 | 2.771 | 29.849 | 0.461 | 2.246 | 6.133 |
| 2011 Register | 74.221 | 3.291 | n.a.^1^ | 0.715 | 2.913 | 6.569 |
| 2011 Survey | 74.888 | 2.980 | n.a.^1^ | 0.573 | 2.334 | 6.410 |

Note: Statistics Austria, EU-SILC 2008-2011. Weighted results. ^1^ n.a. not applicable; in 2011, old-age benefits were already drawn from registers in the primal data collection round.

Table A 4: Over-reporting and Underreporting among different groups, % within group

|  | **survey > register** | **survey < register** | **survey ≈ register** | N (households) |
| --- | --- | --- | --- | --- |
| Epinc, 1^st^ quintile | 49.4% | 21.1% | 29.4% | 1338 |
| Epinc, 2^nd^ quintile | 30.2% | 35.2% | 34.6% | 1178 |
| Epinc, 3^rd^ quintile | 27.0% | 38.4% | 34.6% | 1205 |
| Epinc, 4^th^ quintile | 21.0% | 47.7% | 31.3% | 1205 |
| Epinc, 5^th^ quintile | 15.1% | 58.4% | 26.5% | 1262 |
| Male household head | 29.8% | 39.1% | 31.1% | 2810 |
| Female household head | 28.8% | 39.8% | 31.4% | 3377 |
| Household Head: >6 months Full Time empl. | 30.6% | 39.6% | 29.9% | 2640 |
| Household Head: >6 months Part Time empl. | 28.8% | 41.7% | 29.4% | 667 |
| Household Head: >6 months unemployed | 30.4% | 44.2% | 25.4% | 245 |
| Household Head: >6 months Retired | 26.2% | 38.2% | 35.6% | 2019 |
| Household Head: >6 months Student, School | 42.1% | 27.4% | 30.5% | 157 |
| Household Head: >6 months Housework | 27.2% | 44.0% | 28.8% | 377 |
| Highest Education in Household: basic | 27.9% | 38.6% | 33.4% | 861 |
| Highest Education in Household: middle | 29.6% | 39.2% | 31.2% | 2955 |
| Highest Education in Household: high | 29.6% | 38.4% | 32.0% | 1372 |
| Highest Education in Household: specialized | 29.0% | 42.8% | 28.2% | 1000 |
| Retired household | 24.6% | 39.1% | 36.3% | 1801 |
| Single Person Household not retired | 31.5% | 37.4% | 31.1% | 1197 |
| Multiple Person Household, no children | 31.6% | 42.1% | 26.3% | 1331 |
| Single parent | 26.3% | 34.7% | 39.0% | 306 |
| MPH, children | 31.3% | 40.0% | 28.7% | 1553 |
| Vienna | 30.2% | 37.1% | 32.8% | 1299 |
| >100.000 | 33.3% | 37.5% | 29.2% | 587 |
| >10.000 | 26.6% | 43.0% | 30.4% | 952 |
| <=10.000 | 29.0% | 39.8% | 31.2% | 3350 |
| 0 hh members >15 with >1 employment | 28.9% | 39.7% | 31.4% | 5769 |
| 1 hh member >15 with >1 employment | 34.6% | 37.3% | 28.0% | 400 |
| 2 hh members >15 with >1 employment | 25.0% | 24.8% | 50.1% | 17 |
| 3 hh members >15 with >1 employment | 61.7% | 0.0% | 38.3% | 2 |
| CAPI | 29.7% | 39.4% | 30.8% | 3691 |
| CATI | 28.6% | 39.5% | 31.9% | 2497 |
| Interview month: 3 (March) | 28.5% | 37.1% | 34.3% | 942 |
| Interview month: 4 | 29.8% | 40.2% | 30.0% | 819 |
| Interview month: 5 | 28.8% | 39.4% | 31.8% | 1062 |
| Interview month: 6 | 28.4% | 40.6% | 31.0% | 1408 |
| Interview month: 7 | 26.5% | 40.6% | 32.9% | 849 |
| Interview month: 8 | 31.2% | 38.9% | 29.8% | 592 |
| Interview month: 9 | 34.7% | 40.1% | 25.1% | 395 |
| Interview month: 10 | 39.0% | 34.5% | 26.5% | 98 |
| Interview month: 11 | 23.8% | 35.8% | 40.4% | 23 |
| 0 proxy interviews in hh | 28.6% | 38.6% | 32.8% | 4915 |
| 1 proxy interviews in hh | 32.3% | 42.5% | 25.2% | 1052 |
| 2 proxy interviews in hh | 29.9% | 40.1% | 30.0% | 166 |
| 3 proxy interviews in hh | 21.8% | 54.4% | 23.8% | 44 |
| 4 proxy interviews in hh | 58.1% | 30.4% | 11.5% | 10 |
| 6 proxy interviews in hh | 0.0% | 100.0% | 0.0% | 1 |
| SILC round 1 | 29.6% | 39.8% | 30.6% | 2005 |
| SILC round 2 | 31.3% | 37.8% | 30.9% | 1717 |
| SILC round 3 | 28.2% | 39.3% | 32.5% | 1296 |
| SILC round 4 | 27.1% | 41.5% | 31.4% | 1170 |

Note: Units of observation are households. Weighted results.


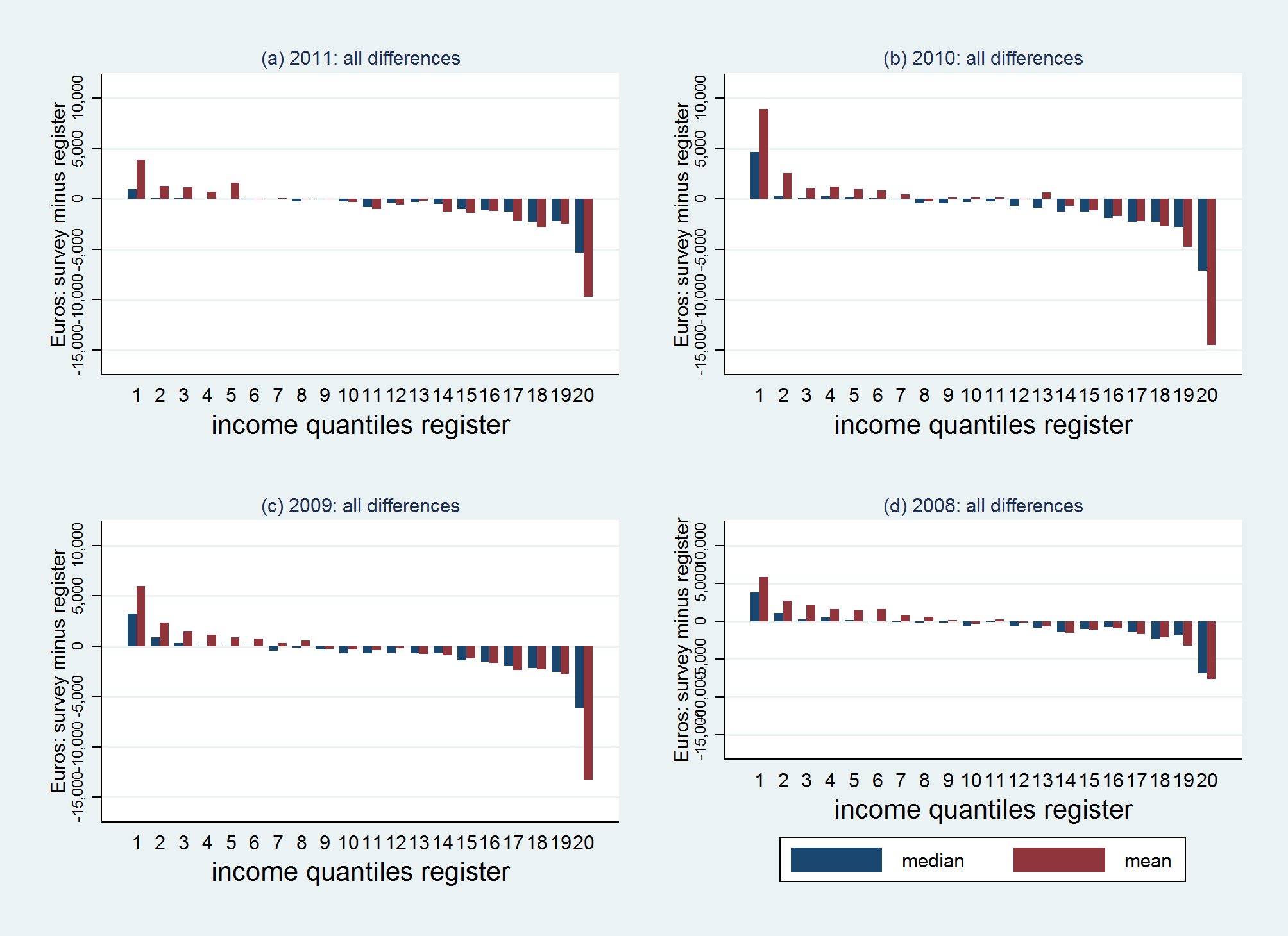


Figure A 1 Median and mean of absolute deviation for equivalised household income for 20 quantiles derived from registers. Weighted data. Persons are units of observation. Income quantiles based on register data. Difference = survey minus register. For logarithmic deviations see Figure A 4.


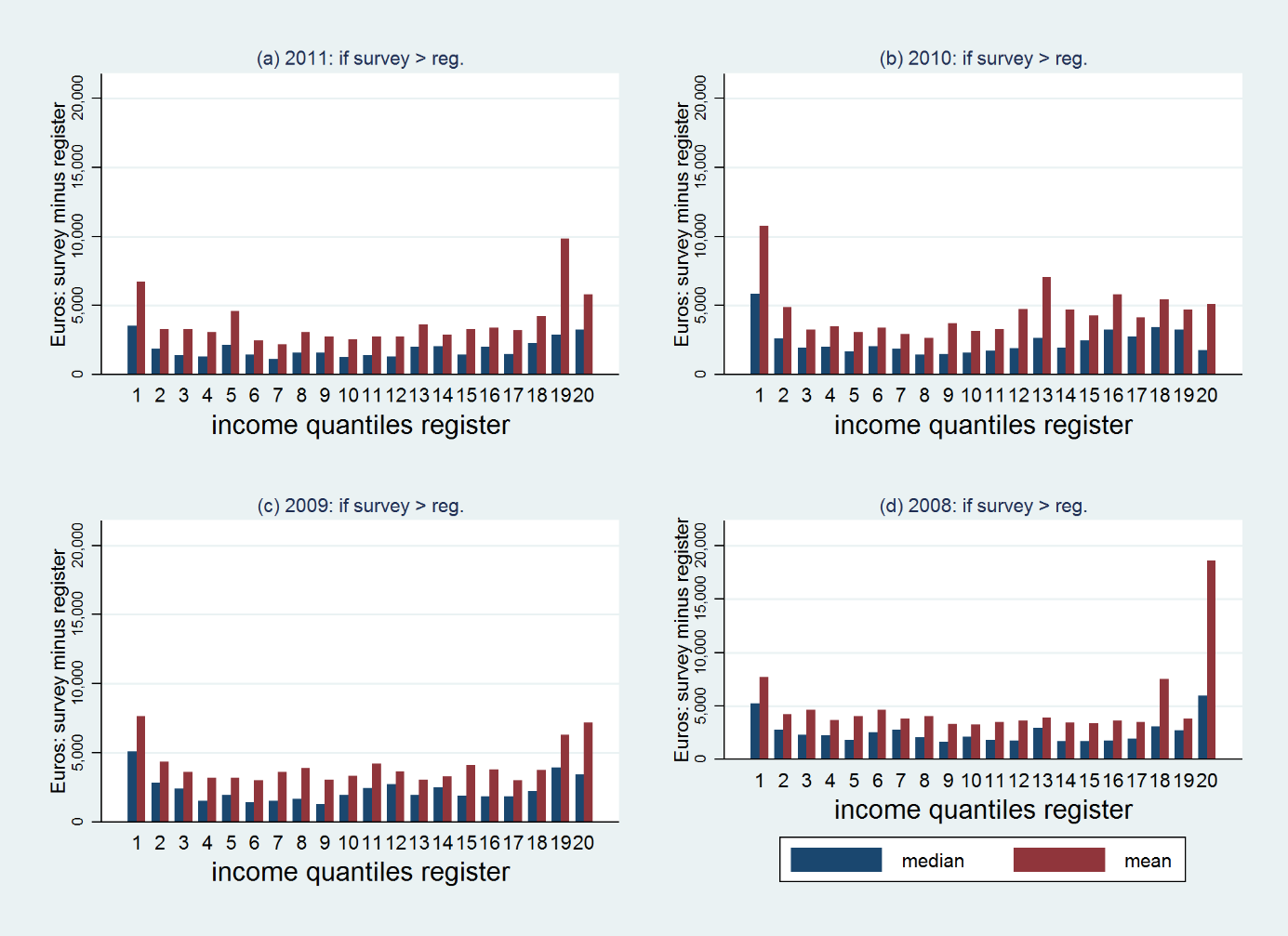


Figure A 2 Median and mean of absolute differences > 0 for equivalised household income for 20 income quantiles derived from registers. Weighted data. Persons are units of observation. Income quantiles based on register data. Difference = survey minus register.


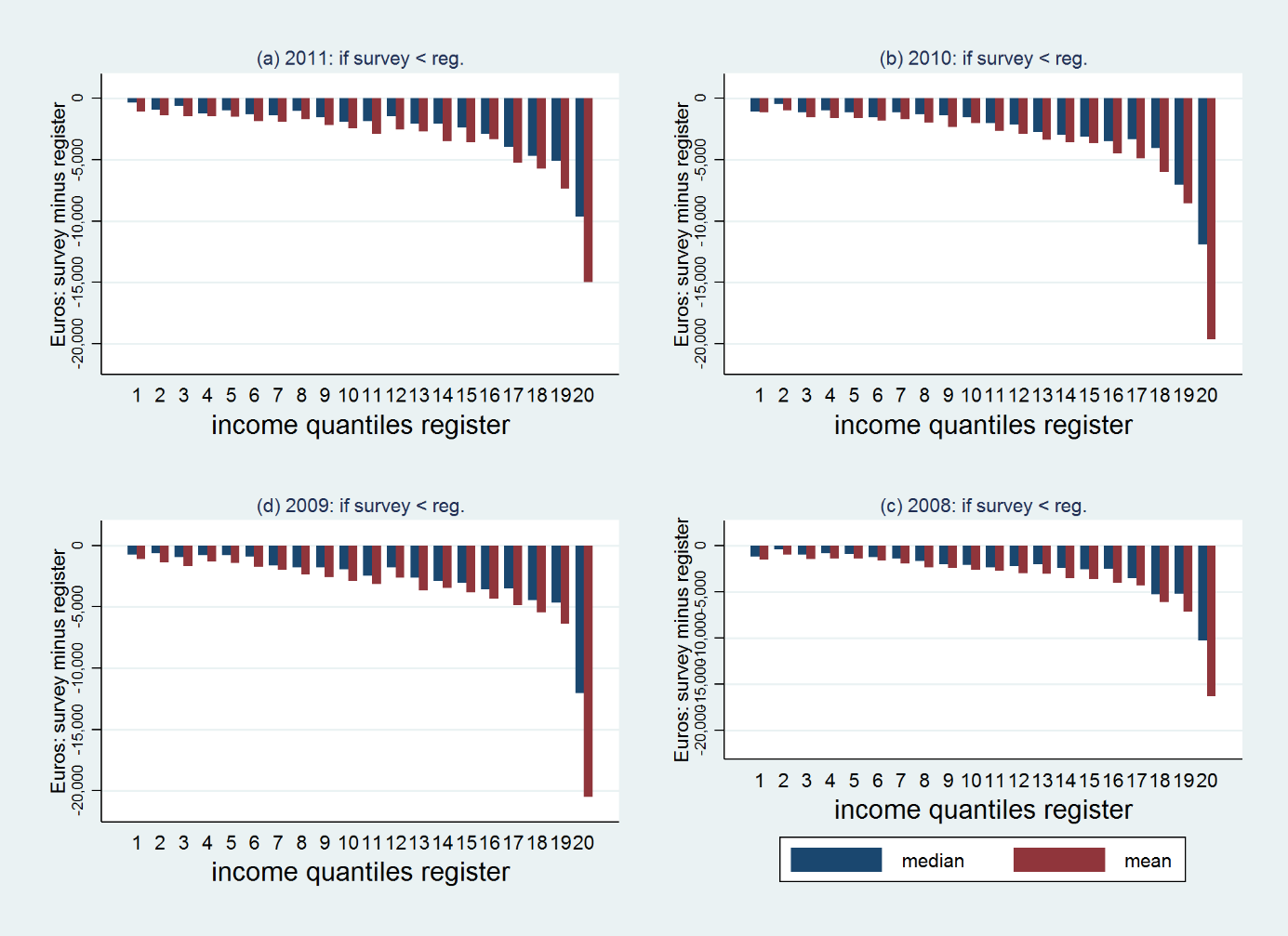


Figure A 3 Median and mean of absolute differences < 0 for equivalised household income for 20 income quantiles derived from registers. Weighted data. Persons are units of observation. Income quantiles based on register data. Difference = survey minus register.


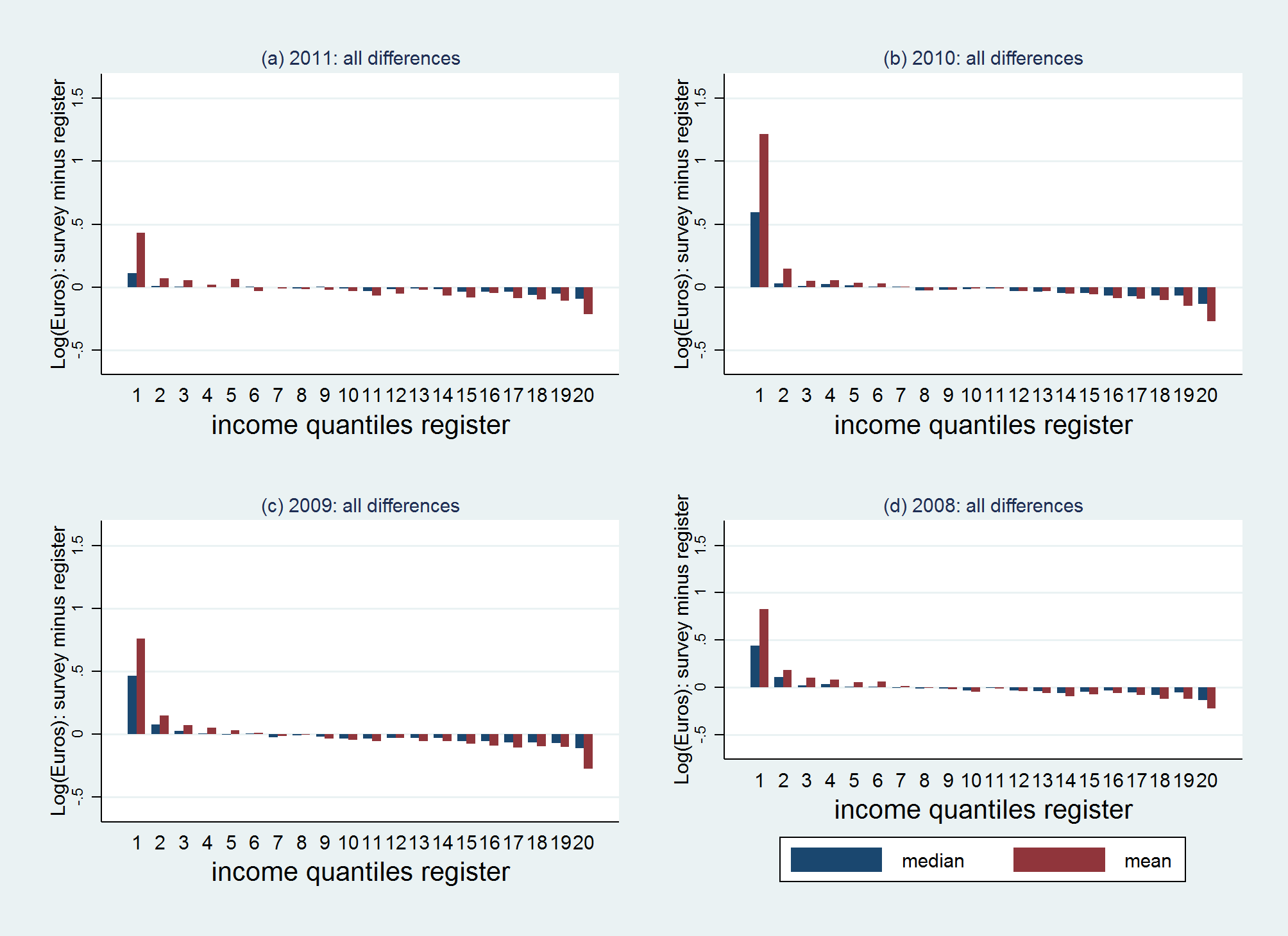


Figure A 4 Median and mean of log differences for equivalised household income for 20 income quantiles derived from registers. Weighted data. Persons are units of observation. Income quantiles based on register data. Difference = survey minus register.

Table A 5 Results from multinomial regression models – odds ratios, EU-SILC 2008 – 2011

| + | **Over-reporting (survey > register)** | | | | **Under-reporting (survey < register)** | | | |
| --- | --- | --- | --- | --- | --- | --- | --- | --- |
|  | **(1)** | **(2)** | **(3)** | **(4)** | **(5)** | **(6)** | **(7)** | **(8)** |
|  | **2008** | **2009** | **2010** | **2011** | **2008** | **2009** | **2010** | **2011** |
| Ln(Epinc) | 0.283^***^ | 0.302^***^ | 0.244^***^ | 0.557^***^ | 3.310^***^ | 2.882^***^ | 3.654^***^ | 3.979^***^ |
| Satisfaction with household income (median)^1^ | 1.222^***^ | 1.234^***^ | 1.228^***^ | 1.099^*^ | 0.888^**^ | 0.887^**^ | 0.830^***^ | 0.837^***^ |
| Main income: employed | 3.622 | 0.697 | 1.340 | 1.324 | 1.233 | 0.742 | 1.112 | 1.013 |
| Main income: self-employment | 1.357 | 0.494 | 0.790 | 0.631 | 0.406^*^ | 0.207^**^ | 0.239^***^ | 0.289^**^ |
| Main income: social transfers | 3.009 | 0.708 | 1.049 | 1.433 | 1.338 | 0.633 | 1.162 | 0.953 |
| Main income: old-age benefits | Ref. cat. | Ref. cat. | Ref. cat. | Ref. cat. | Ref. cat. | Ref. cat. | Ref. cat. | Ref. cat. |
| Main income: other private | 2.652 | 0.425 | 0.984 | 0.828 | 0.128^***^ | 0.199^**^ | 0.0892^***^ | 0.0728^***^ |
| Total no. of different income components | 0.991 | 1.011 | 1.034 | 1.139^**^ | 1.008 | 0.998 | 1.026 | 1.098^*^ |
| Activity status^2^: full time work | Ref. cat. | Ref. cat. | Ref. cat. | Ref. cat. | Ref. cat. | Ref. cat. | Ref. cat. | Ref. cat. |
| Activity status: part time work | 0.809 | 0.849 | 0.845 | 1.435^*^ | 1.145 | 0.948 | 1.190 | 1.418^*^ |
| Activity status: unemployed | 0.829 | 0.688 | 0.682 | 0.829 | 1.280 | 1.724^**^ | 1.817^**^ | 1.418 |
| Activity status: retired | 0.897 | 0.742 | 0.849 | 0.719 | 1.069 | 0.821 | 0.949 | 0.909 |
| Activity status: student, other | 0.298^**^ | 0.658 | 0.462^**^ | 1.142 | 2.807^*^ | 1.446 | 1.383 | 3.200^***^ |
| Activity status: housework | 0.597^**^ | 1.070 | 0.778 | 1.334 | 1.516^**^ | 1.620^**^ | 1.610^**^ | 1.896^***^ |
| No. hh members >15 with >1 employment | 1.660^**^ | 1.250 | 1.258 | 1.010 | 1.088 | 1.114 | 0.809 | 0.725^*^ |
| Employ. status changes: none | Ref. cat. | Ref. cat. | Ref. cat. | Ref. cat. | Ref. cat. | Ref. cat. | Ref. cat. | Ref. cat. |
| Employ. status changes: 1 | 1.300^*^ | 1.459^**^ | 1.464^***^ | 1.415^**^ | 1.332^*^ | 1.407^**^ | 1.383^**^ | 1.279^*^ |
| Employ. status changes: >1 | 1.407 | 1.299 | 1.843^*^ | 1.210 | 1.333 | 1.311 | 1.794^*^ | 1.204 |
| Age | 0.938^***^ | 0.953^**^ | 0.968^*^ | 1.047^*^ | 0.953^**^ | 0.978 | 1.001 | 1.111^***^ |
| Age squared | 1.001^***^ | 1.000^***^ | 1.000^*^ | 0.999^***^ | 1.000^**^ | 1.000^*^ | 1.000 | 0.999^***^ |
| Male | Ref. cat. | Ref. cat. | Ref. cat. | Ref. cat. | Ref. cat. | Ref. cat. | Ref. cat. | Ref. cat. |
| Female | 0.931 | 0.870 | 0.992 | 0.693^***^ | 1.150 | 1.060 | 1.056 | 0.850 |
| Household sickness (Median)^3^ | 1.077 | 1.015 | 0.978 | 0.967 | 1.146^**^ | 1.105^*^ | 1.062 | 1.073 |
| Education: basic | Ref. cat. | Ref. cat. | Ref. cat. | Ref. cat. | Ref. cat. | Ref. cat. | Ref. cat. | Ref. cat. |
| Education: middle | 1.173 | 1.402^**^ | 1.415^**^ | 1.206 | 0.755^*^ | 0.757^*^ | 0.874 | 0.810 |
| Education: high | 1.564^**^ | 1.683^***^ | 1.419^*^ | 1.303 | 0.654^**^ | 0.778 | 0.707^*^ | 0.771 |
| Education: specialized | 1.534^*^ | 1.803^***^ | 1.755^***^ | 1.236 | 0.699^*^ | 0.782 | 0.805 | 0.691^*^ |
| Retired household | 1.617 | 0.392 | 0.678 | 0.548 | 0.620 | 0.487 | 0.921 | 0.429^*^ |
| Single HH, not retired | 0.669^**^ | 0.651^**^ | 0.652^**^ | 0.734^*^ | 0.830 | 0.778 | 1.026 | 0.906 |
| MPH, no children | Ref. cat. | Ref. cat. | Ref. cat. | Ref. cat. | Ref. cat. | Ref. cat. | Ref. cat. | Ref. cat. |
| Single parent | 0.429^***^ | 0.402^***^ | 0.393^***^ | 0.362^***^ | 0.803 | 0.729 | 0.803 | 0.667^*^ |
| MPH, children | 0.702^**^ | 0.729^*^ | 0.627^***^ | 0.653^***^ | 1.156 | 1.075 | 1.066 | 1.084 |
| Region: Vienna (capital, >1,000,000 inh.) | Ref. cat. | Ref. cat. | Ref. cat. | Ref. cat. | Ref. cat. | Ref. cat. | Ref. cat. | Ref. cat. |
| >100,000 inhabitants | 1.151 | 0.739 | 1.113 | 0.844 | 1.463^*^ | 0.980 | 1.171 | 0.951 |
| >10,000 inhabitants | 0.725^*^ | 0.979 | 1.011 | 0.716^*^ | 1.082 | 1.138 | 1.205 | 1.002 |
| <=10,000 inhabitants | 1.007 | 0.894 | 1.045 | 0.785^*^ | 1.257^*^ | 1.022 | 1.129 | 1.038 |
| CAPI | Ref. cat. | Ref. cat. | Ref. cat. | Ref. cat. | Ref. cat. | Ref. cat. | Ref. cat. | Ref. cat. |
| CATI | 0.982 | 1.131 | 1.046 | 0.901 | 0.926 | 0.893 | 0.936 | 0.668^***^ |
| Sum of proxy interviews in household | 1.118 | 1.065 | 1.199^*^ | 1.038 | 1.041 | 1.080 | 1.159^*^ | 1.101 |
| Interview month | 1.018 | 1.055 | 1.030 | 1.032 | 1.071^*^ | 1.071^*^ | 1.036 | 0.970 |
| SILC round 1 | Ref. cat. | Ref. cat. | Ref. cat. | Ref. cat. | Ref. cat. | Ref. cat. | Ref. cat. | Ref. cat. |
| SILC round 2 | 1.221 | 0.794 | 0.999 | 1.127 | 1.009 | 1.027 | 1.002 | 1.130 |
| SILC round 3 | 1.064 | 0.815 | 0.896 | 1.022 | 1.061 | 0.977 | 1.008 | 1.010 |
| SILC round 4 | 1.050 | 0.986 | 0.858 | 0.890 | 1.005 | 1.132 | 1.079 | 1.007 |
| N (Households) | 5629 | 5802 | 6074 | 6070 | 5629 | 5802 | 6074 | 6070 |

Note: * p < 0.05, ** p < 0.01, *** p < 0.001. ^1^ scale from 1 to 6; 1=very unhappy, 6= very happy. ^2^ Self-reported labor status in income reference period for 2010 for more than 6 months. ^3^ scale from 1 to 5; 1=very good 5 = very bad.

*Multinomial logit regression models* using sampling weights: Coefficients of the model are estimated at once. Sample size in col. 1,2,3,4 and 5,6,7,8 refers to the sum over all 3 categories of the dependent variable. Coefficients show odds ratios. Odds For the dependent variable, the reference category refers to households with a difference between equivalised household incomes that lies within the range of +/- 5%. Standard errors (not displayed) account for complex stratified survey design. Pseudo R^2 measures are not available for Maximum Likelihood estimation as the assumption of observations being independent and identically distributed (*iid*) is not fulfilled. MPH: Multiple person household. Epinc was used in log form after comparing the Akaike and Bayesian Information Criteria.

Table A 6: Results from regression models – OLS, dependent variable is epincdelta (survey minus register)

|  | **Over-reporting (survey > register)** | | | | **Under-reporting (survey < register)** | | | |
| --- | --- | --- | --- | --- | --- | --- | --- | --- |
|  | **(1)** | **(2)** | **(3)** | **(4)** | **(5)** | **(6)** | **(7)** | **(8)** |
|  | **2008** | **2009** | **2010** | **2011** | **2008** | **2009** | **2010** | **2011** |
| Epinc | 0.00536 | -0.0332 | -0.299^***^ | -0.155^**^ | 0.177^***^ | 0.183^**^ | 0.223^***^ | 0.257^***^ |
| Epinc squared |  |  | 0.00000204^***^ | 0.00000117^*^ | 0.00000206^***^ | 0.00000211^**^ | 0.00000194^***^ |  |
| Satisfaction with household income (median)^1^ | 526.9^***^ | 510.6^***^ | 981.1^***^ | 868.6^***^ | -338.3^***^ | -427.2^**^ | -942.1^***^ | -488.2^**^ |
| Main income: employed | 2530.0^*^ | -1294.3 | 2894.4^***^ | -1438.6 | 2065.7^*^ | 45.18 | 853.2 | 450.3 |
| Main income: self-employment | 1740.9 | 43.03 | 2217.6^*^ | -2528.1 | -523.0 | -4343.8^***^ | -1966.1 | -1914.6^*^ |
| Main income: social transfers | 4063.9^***^ | -161.6 | 2786.0^***^ | -114.1 | 1865.5^*^ | 130.5 | 738.7 | 183.8 |
| Main income: old-age benefits | Ref. cat. | Ref. cat. | Ref. cat. | Ref. cat. | Ref. cat. | Ref. cat. | Ref. cat. | Ref. cat. |
| Main income: other private | 9206.5^***^ | 4974.5^**^ | 9869.8^***^ | 2897.6 | -7648.5^**^ | -4792.0^**^ | -8900.5^**^ | -6147.7 |
| Total no. of different income components | -679.1^**^ | -648.1^***^ | -377.9^*^ | -602.7^***^ | -344.7^***^ | -303.1^**^ | -292.7^*^ | -301.5^*^ |
| Activity status^2^: full time work | Ref. cat. | Ref. cat. | Ref. cat. | Ref. cat. | Ref. cat. | Ref. cat. | Ref. cat. | Ref. cat. |
| Activity status: part time work | 1624.4 | -1313.7^***^ | -742.7 | -820.5 | 1140.0^**^ | 815.3 | 381.4 | 1369.5^**^ |
| Activity status: unemployed | -1037.2 | -1682.4^**^ | -3039.4^***^ | -2301.3^***^ | 1344.8^*^ | 1749.7^**^ | 1133.3^**^ | 1414.6^**^ |
| Activity status: retired | 672.0 | -1182.0 | -1076.0 | -2196.6^**^ | 619.8 | 52.96 | 137.2 | 90.08 |
| Activity status: student, other | -3333.7 | -2545.9^*^ | -5359.2^***^ | -5041.0^***^ | 1929.5^*^ | 2004.3^**^ | 2218.5^**^ | 3033.2^***^ |
| Activity status: housework | -453.1 | -1255.1^*^ | -1748.5^*^ | -2383.8^**^ | 1396.7^***^ | 1912.0^**^ | 1488.1^**^ | 1489.0^**^ |
| No. hh members >15 with >1 employment | 1319.1 | 174.8 | -159.0 | -107.0 | -1018.4^*^ | -994.3 | -1745.5^***^ | -793.4 |
| Employ. status changes: none | Ref. cat. | Ref. cat. | Ref. cat. | Ref. cat. | Ref. cat. | Ref. cat. | Ref. cat. | Ref. cat. |
| Employ. status changes: 1 | -134.1 | 859.6^*^ | 6.807 | -538.5 | 815.4^*^ | 933.8^*^ | 727.4^*^ | 640.6^*^ |
| Employ. status changes: >1 | 36.53 | 902.8 | -174.2 | -583.6 | 1587.7^*^ | 759.9 | 817.6 | 656.9 |
| Age | 33.57 | -72.62 | 45.93^**^ | -11.71 | -141.8^***^ | -27.00 | -20.04 | 78.98^**^ |
| Age squared |  | 1.188^**^ |  |  | 1.149^***^ |  |  | -0.839^**^ |
| Male | Ref. cat. | Ref. cat. | Ref. cat. | Ref. cat. | Ref. cat. | Ref. cat. | Ref. cat. | Ref. cat. |
| Female | -777.3 | 56.87 | 85.31 | -130.3 | -244.6 | -142.2 | -7.855 | -422.5 |
| Household sickness (Median)^3^ | -206.4 | 15.42 | -80.02 | -117.5 | -80.52 | 42.28 | 68.11 | 277.1^*^ |
| Education: basic | Ref. cat. | Ref. cat. | Ref. cat. | Ref. cat. | Ref. cat. | Ref. cat. | Ref. cat. | Ref. cat. |
| Education: middle | 464.2 | 1229.8^***^ | 1104.7^**^ | 1047.0^**^ | -782.3^**^ | -365.6 | -643.3 | -291.6 |
| Education: high | 1469.8^*^ | 1637.7^***^ | 1750.6^***^ | 1434.5^**^ | -1199.2^***^ | -1392.4^***^ | -1884.3^***^ | -489.3 |
| Education: specialized | 4426.8^***^ | 3277.4^***^ | 4251.6^***^ | 3817.3^***^ | -1825.4^***^ | -1745.6^***^ | -2871.6^***^ | -1269.8^*^ |
| Retired household | 933.0 | -2411.6 | 82.35 | -2717.3 | 948.9 | 827.5 | 1391.5 | -488.6 |
| Single HH, not retired | -866.9 | 77.76 | -1891.1^*^ | -1357.3 | -437.0 | 408.0 | 1485.8^**^ | 382.7 |
| MPH, no children | Ref. cat. | Ref. cat. | Ref. cat. | Ref. cat. | Ref. cat. | Ref. cat. | Ref. cat. | Ref. cat. |
| Single parent | -1164.6 | -1134.4 | -2706.5^**^ | -2338.6^**^ | -122.4 | 1582.2 | 1562.5^*^ | 901.1 |
| MPH, children | -412.5 | -733.9 | -2138.6^**^ | -1340.6^*^ | 372.5 | 1078.9^*^ | 1634.3^***^ | 958.3^*^ |
| Region: Vienna (capital, >1,000,000 inh.) | Ref. cat. | Ref. cat. | Ref. cat. | Ref. cat. | Ref. cat. | Ref. cat. | Ref. cat. | Ref. cat. |
| >100,000 inhabitants | -179.8 | -778.7 | -491.2 | -67.66 | -236.1 | -187.0 | 122.8 | 83.19 |
| >10,000 inhabitants | 27.23 | -117.7 | -354.4 | -45.64 | 25.52 | 151.7 | -387.5 | 256.8 |
| <=10,000 inhabitants | 87.14 | -557.2 | -1003.0^*^ | -313.4 | -342.0 | -125.8 | 187.3 | 38.19 |
| CAPI | Ref. cat. | Ref. cat. | Ref. cat. | Ref. cat. | Ref. cat. | Ref. cat. | Ref. cat. | Ref. cat. |
| CATI | -203.7 | 295.7 | 482.2 | -30.50 | -552.8^*^ | -392.6 | -325.9 | -674.6^*^ |
| Sum of proxy interviews in hh | -3.365 | 175.1 | 12.81 | 23.29 | -48.27 | 96.35 | 139.6 | 424.8^*^ |
| Interview month | 110.5 | 101.9 | -129.9 | -54.57 | 46.49 | 1.958 | 164.2^*^ | -134.0 |
| SILC round 1 | Ref. cat. | Ref. cat. | Ref. cat. | Ref. cat. | Ref. cat. | Ref. cat. | Ref. cat. | Ref. cat. |
| SILC round 2 | 477.1 | -626.7 | -637.2 | -71.85 | -187.5 | 299.0 | -477.2 | 470.9 |
| SILC round 3 | -228.8 | -671.2 | -2255.7^***^ | -787.6 | 437.9 | 177.8 | -36.69 | 611.9 |
| SILC round 4 | -367.6 | -770.2 | -1597.9^***^ | -1096.9^**^ | 254.2 | 1104.9 | -219.8 | 468.9 |
| Constant | -560.5 | 5697.4^**^ | 4592.0^*^ | 8518.5^***^ | 3781.9^*^ | 1828.5 | 1231.5 | -2229.5 |
| R^2^ | 0.056 | 0.099 | 0.166 | 0.091 | 0.636 | 0.573 | 0.689 | 0.334 |
| N (Households) | 2451 | 2505 | 2448 | 2268 | 3098 | 3238 | 3546 | 3192 |

Note: * p < 0.05, ** p < 0.01, *** p < 0.001. ^1^ scale from 1 to 6; 1=very unhappy, 6= very happy. ^2^ Self-reported labor status in income reference period for 2010 for more than 6 months. ^3^ scale from 1 to 5; 1=very good 5 = very bad.

*OLS regression models* using sampling weights: Dependent variable is epincdelta (survey minus register). Standard errors (not displayed) account for complex survey design (strata=federal states). *Age squared* and *epinc squared* where only included in the model if the Wald test was significant.

Table A 7: OLS regression models. Dependent variable measured in logs, all years 2008-2011

|  | **survey > register** | | | | **survey < register** | | | |
| --- | --- | --- | --- | --- | --- | --- | --- | --- |
|  | **(1)** | **(2)** | **(3)** | **(4)** | **(5)** | **(6)** | **(7)** | **(8)** |
|  | **2008** | **2009** | **2010** | **2011** | **2008** | **2009** | **2010** | **2011** |
| Ln(Epinc) | -0.372^***^ | -0.354^***^ | -0.489^***^ | -0.553^***^ | 1.749^***^ | 1.632^***^ | 1.983^***^ | 1.833^***^ |
| Satisfaction with household income (median)^1^ | 0.149^***^ | 0.167^***^ | 0.220^***^ | 0.147^***^ | -0.0628^*^ | -0.0655^*^ | -0.156^***^ | -0.166^***^ |
| Main income: employed | 0.614 | -0.563 | 0.672^*^ | -0.225 | 0.466 | 0.0189 | -0.258 | -0.477 |
| Main income: self-employment | 0.376 | -0.396 | 0.591 | -0.712 | -0.0286 | -0.506 | -0.823^***^ | -1.125^***^ |
| Main income: social transfers | 0.734 | -0.477 | 0.531 | -0.591 | 0.612^*^ | 0.0776 | -0.0332 | -0.816^**^ |
| Main income: old-age benefits | Ref. cat. | Ref. cat. | Ref. cat. | Ref. cat. | Ref. cat. | Ref. cat. | Ref. cat. | Ref. cat. |
| Main income: other private | 1.091^*^ | -0.354 | 0.727^*^ | -0.0875 | -0.855^*^ | -0.790 | -1.801^***^ | -1.679^**^ |
| Total no. of different income components | -0.0871^*^ | -0.0803^*^ | -0.0345 | -0.00397 | -0.0352 | -0.0313 | -0.0149 | 0.0292 |
| Activity status^2^: full time work | Ref. cat. | Ref. cat. | Ref. cat. | Ref. cat. | Ref. cat. | Ref. cat. | Ref. cat. | Ref. cat. |
| Activity status: part time work | -0.0373 | -0.202 | -0.0647 | 0.321^**^ | 0.198^*^ | 0.0451 | 0.0129 | 0.311^***^ |
| Activity status: unemployed | -0.435^*^ | -0.341 | -0.392^*^ | -0.259 | 0.143 | 0.713^***^ | 0.331^*^ | 0.850^***^ |
| Activity status: retired | -0.240 | -0.324^*^ | -0.0826 | -0.547^*^ | -0.0755 | -0.124 | -0.174 | -0.136 |
| Activity status: student, other | -0.870^**^ | -0.382 | -0.426^*^ | -0.0936 | 0.940^***^ | 0.382 | 0.569^**^ | 1.250^***^ |
| Activity status: housework | -0.312^*^ | -0.259 | -0.234 | 0.199 | 0.258^*^ | 0.359^**^ | 0.274^*^ | 0.794^***^ |
| No. hh members >15 with >1 employment | 0.222^*^ | -0.00330 | 0.0395 | 0.0356 | -0.0673 | 0.0280 | -0.154 | -0.198^*^ |
| Employ. status changes: none | Ref. cat. | Ref. cat. | Ref. cat. | Ref. cat. | Ref. cat. | Ref. cat. | Ref. cat. | Ref. cat. |
| Employ. status changes: 1 | 0.0720 | 0.250^**^ | 0.221^**^ | 0.0775 | 0.178^*^ | 0.182^*^ | 0.147^*^ | 0.270^***^ |
| Employ. status changes: >1 | -0.184 | 0.337 | 0.462^**^ | 0.186 | 0.396^**^ | 0.0789 | 0.295^*^ | 0.245 |
| Age | -0.0268^*^ | -0.0219^*^ | 0.00202 | 0.124^***^ | -0.0367^***^ | -0.0287^**^ | -0.0169 | 0.129^***^ |
| Age squared | 0.000284^*^ | 0.000273^*^ |  | -0.00162^***^ | 0.000357^***^ | 0.000290^***^ | 0.000179^*^ | -0.00161^***^ |
| Male | Ref. cat. | Ref. cat. | Ref. cat. | Ref. cat. | Ref. cat. | Ref. cat. | Ref. cat. | Ref. cat. |
| Female | -0.0580 | -0.0379 | 0.0234 | -0.435^***^ | -0.00488 | -0.0502 | 0.0249 | -0.369^***^ |
| Household sickness (Median)^3^ | 0.0448 | 0.0969^*^ | 0.00324 | 0.0389 | 0.0629 | 0.0263 | 0.0323 | 0.0696 |
| Education: basic | Ref. cat. | Ref. cat. | Ref. cat. | Ref. cat. | Ref. cat. | Ref. cat. | Ref. cat. | Ref. cat. |
| Education: middle | 0.267^*^ | 0.403^***^ | 0.338^**^ | 0.717^***^ | -0.220^*^ | -0.239^**^ | -0.119 | 0.0715 |
| Education: high | 0.434^**^ | 0.636^***^ | 0.437^***^ | 0.841^***^ | -0.187 | -0.271^**^ | -0.346^***^ | -0.00125 |
| Education: specialized | 0.649^***^ | 0.727^***^ | 0.683^***^ | 1.318^***^ | -0.228 | -0.270^*^ | -0.275^**^ | -0.0758 |
| Retired household | 0.230 | -1.040^**^ | 0.156 | -0.653 | -0.0329 | -0.156 | -0.281 | -1.146^***^ |
| Single HH, not retired | -0.169 | -0.128 | -0.312^**^ | -0.423^**^ | -0.0663 | -0.0254 | 0.195^**^ | -0.0575 |
| MPH, no children | Ref. cat. | Ref. cat. | Ref. cat. | Ref. cat. | Ref. cat. | Ref. cat. | Ref. cat. | Ref. cat. |
| Single parent | -0.476^*^ | -1.030^***^ | -0.740^***^ | -0.851^***^ | -0.167 | 0.0355 | 0.0734 | -0.304^*^ |
| MPH, children | -0.237^*^ | -0.304^**^ | -0.475^***^ | -0.612^***^ | 0.0675 | 0.0228 | 0.196^**^ | -0.0757 |
| Region: Vienna (capital, >1,000,000 inh.) | Ref. cat. | Ref. cat. | Ref. cat. | Ref. cat. | Ref. cat. | Ref. cat. | Ref. cat. | Ref. cat. |
| >100,000 inhabitants | 0.186 | -0.171 | -0.0374 | 0.111 | 0.0389 | 0.0967 | 0.138 | 0.210 |
| >10,000 inhabitants | -0.126 | 0.0331 | -0.128 | 0.0171 | 0.00517 | 0.0863 | 0.0374 | 0.0491 |
| <=10,000 inhabitants | -0.0555 | -0.0325 | -0.139 | 0.00308 | 0.0796 | 0.0119 | 0.159^**^ | 0.0551 |
| CAPI | Ref. cat. | Ref. cat. | Ref. cat. | Ref. cat. | Ref. cat. | Ref. cat. | Ref. cat. | Ref. cat. |
| CATI | 0.0640 | 0.0254 | 0.142 | -0.166 | -0.160^*^ | -0.0392 | -0.0562 | -0.330^***^ |
| Sum of proxy interviews in household | 0.0767 | 0.0255 | 0.0871 | 0.115 | 0.00273 | 0.0516 | 0.0795 | 0.198^***^ |
| Interview month | 0.0336 | 0.0681^**^ | 0.00267 | 0.0251 | 0.00523 | 0.0428^*^ | 0.0216 | -0.0350 |
| SILC round 1 | Ref. cat. | Ref. cat. | Ref. cat. | Ref. cat. | Ref. cat. | Ref. cat. | Ref. cat. | Ref. cat. |
| SILC round 2 | 0.146 | -0.178 | -0.0895 | 0.109 | -0.0373 | -0.0188 | -0.111 | 0.240^*^ |
| SILC round 3 | -0.0605 | -0.113 | -0.357^***^ | -0.110 | 0.0691 | -0.0452 | -0.0338 | 0.125 |
| SILC round 4 | 0.0905 | -0.0566 | -0.204 | -0.197 | -0.0160 | 0.0405 | -0.111 | 0.177 |
| Constant | 10.39^***^ | 10.75^***^ | 10.83^***^ | 10.04^***^ | -9.105^***^ | -7.897^***^ | -11.25^***^ | -12.29^***^ |
| N (Households) | 2450 | 2503 | 2448 | 2264 | 3098 | 3238 | 3546 | 3192 |
| R^2^ | 0.101 | 0.114 | 0.146 | 0.314 | 0.281 | 0.263 | 0.345 | 0.377 |

Note: * p < 0.05, ** p < 0.01, *** p < 0.001. ^1^ scale from 1 to 6; 1=very unhappy, 6= very happy. ^2^ Self-reported labor status in income reference period for 2010 for more than 6 months. ^3^ scale from 1 to 5; 1=very good 5 = very bad.

*OLS regression models* using sampling weights: Dependent variable is epincdelta (survey minus register). Standard errors (not displayed) account for complex survey design (strata=federal states). *Age squared* and *epinc squared* where only included in the model if the Wald test was significant.

Table A 8: OLS models. Level-log specification for income difference (>0) and income on right hand side

|  | **survey > register** | | | |
| --- | --- | --- | --- | --- |
|  | **(1)** | **(2)** | **(3)** | **(4)** |
|  | **2008** | **2009** | **2010** | **2011** |
| Ln(Epinc) | -0.372^***^ | -0.354^***^ | -0.489^***^ | -0.553^***^ |
| Satisfaction with household income (median)^1^ | 0.149^***^ | 0.167^***^ | 0.220^***^ | 0.147^***^ |
| Main income: employed | 0.614 | -0.563 | 0.672^*^ | -0.225 |
| Main income: self-employment | 0.376 | -0.396 | 0.591 | -0.712 |
| Main income: social transfers | 0.734 | -0.477 | 0.531 | -0.591 |
| Main income: old-age benefits | 0 | 0 | 0 | 0 |
| Main income: other private | 1.091^*^ | -0.354 | 0.727^*^ | -0.0875 |
| Total no. of different income components | -0.0871^*^ | -0.0803^*^ | -0.0345 | -0.00397 |
| Activity status^2^: full time work | Ref. cat. | Ref. cat. | Ref. cat. | Ref. cat. |
| Activity status: part time work | -0.0373 | -0.202 | -0.0647 | 0.321^**^ |
| Activity status: unemployed | -0.435^*^ | -0.341 | -0.392^*^ | -0.259 |
| Activity status: retired | -0.240 | -0.324^*^ | -0.0826 | -0.547^*^ |
| Activity status: student, other | -0.870^**^ | -0.382 | -0.426^*^ | -0.0936 |
| Activity status: housework | -0.312^*^ | -0.259 | -0.234 | 0.199 |
| No. household members >15 with >1 employment | 0.222^*^ | -0.00330 | 0.0395 | 0.0356 |
| Employ. status changes: none | Ref. cat. | Ref. cat. | Ref. cat. | Ref. cat. |
| Employ. status changes: 1 | 0.0720 | 0.250^**^ | 0.221^**^ | 0.0775 |
| Employ. status changes: >1 | -0.184 | 0.337 | 0.462^**^ | 0.186 |
| Age | -0.0268^*^ | -0.0219^*^ | 0.00202 | 0.124^***^ |
| Age squared | 0.000284^*^ | 0.000273^*^ |  | -0.00162^***^ |
| Male | Ref. cat. | Ref. cat. | Ref. cat. | Ref. cat. |
| Female | -0.0580 | -0.0379 | 0.0234 | -0.435^***^ |
| Household sickness (Median)^3^ | 0.0448 | 0.0969^*^ | 0.00324 | 0.0389 |
| Education: basic | Ref. cat. | Ref. cat. | Ref. cat. | Ref. cat. |
| Education: middle | 0.267^*^ | 0.403^***^ | 0.338^**^ | 0.717^***^ |
| Education: high | 0.434^**^ | 0.636^***^ | 0.437^***^ | 0.841^***^ |
| Education: specialized | 0.649^***^ | 0.727^***^ | 0.683^***^ | 1.318^***^ |
| Retired household | 0.230 | -1.040^**^ | 0.156 | -0.653 |
| Single HH, not retired | -0.169 | -0.128 | -0.312^**^ | -0.423^**^ |
| MPH, no children | Ref. cat. | Ref. cat. | Ref. cat. | Ref. cat. |
| Single parent | -0.476^*^ | -1.030^***^ | -0.740^***^ | -0.851^***^ |
| MPH, children | -0.237^*^ | -0.304^**^ | -0.475^***^ | -0.612^***^ |
| Region: Vienna (capital, >1,000,000 inhabitants) | Ref. cat. | Ref. cat. | Ref. cat. | Ref. cat. |
| >100,000 inhabitants | 0.186 | -0.171 | -0.0374 | 0.111 |
| >10,000 inhabitants | -0.126 | 0.0331 | -0.128 | 0.0171 |
| <=10,000 inhabitants | -0.0555 | -0.0325 | -0.139 | 0.00308 |
| CAPI | Ref. cat. | Ref. cat. | Ref. cat. | Ref. cat. |
| CATI | 0.0640 | 0.0254 | 0.142 | -0.166 |
| Sum of proxy interviews in household | 0.0767 | 0.0255 | 0.0871 | 0.115 |
| Interview month | 0.0336 | 0.0681^**^ | 0.00267 | 0.0251 |
| SILC round 1 | Ref. cat. | Ref. cat. | Ref. cat. | Ref. cat. |
| SILC round 2 | 0.146 | -0.178 | -0.0895 | 0.109 |
| SILC round 3 | -0.0605 | -0.113 | -0.357^***^ | -0.110 |
| SILC round 4 | 0.0905 | -0.0566 | -0.204 | -0.197 |
| Constant | 10.39^***^ | 10.75^***^ | 10.83^***^ | 10.04^***^ |
| N (Households) | 2450 | 2503 | 2448 | 2264 |
| R^2^ | 0.101 | 0.114 | 0.146 | 0.314 |

Note: * p < 0.05, ** p < 0.01, *** p < 0.001. ^1^ scale from 1 to 6; 1=very unhappy, 6= very happy. ^2^ Self-reported labor status in income reference period for 2010 for more than 6 months. ^3^ scale from 1 to 5; 1=very good 5 = very bad.

*OLS regression models* using sampling weights: Dependent variable is epincdelta (survey minus register). Standard errors (not displayed) account for complex survey design (strata=federal states). *Age squared* and *epinc squared* where only included in the model if the Wald test was significant.

Table A 9: Poisson Regression Models

|  | **survey > register** | | | | **survey < register** | | | |
| --- | --- | --- | --- | --- | --- | --- | --- | --- |
|  | **(1)** | **(2)** | **(3)** | **(4)** | **(5)** | **(6)** | **(7)** | **(8)** |
|  | **2008** | **2009** | **2010** | **2011** | **2008** | **2009** | **2010** | **2011** |
| Ln(Epinc) | -0.161^**^ | -0.193^***^ | -0.272^***^ | -0.278^***^ | 1.802^***^ | 1.861^***^ | 1.872^***^ | 1.820^***^ |
| Satisfaction with household income (median)^1^ | 0.132^***^ | 0.130^***^ | 0.166^***^ | 0.212^***^ | -0.0810^***^ | -0.105^***^ | -0.154^***^ | -0.134^***^ |
| Main income: employed | 0.642^*^ | -0.261 | 0.691^**^ | -0.308 | 0.370^*^ | -0.000343 | 0.129 | 0.00393 |
| Main income: self-employment | 0.491 | 0.0157 | 0.609^**^ | -0.508 | -0.253 | -0.942^***^ | -0.410 | -0.753^**^ |
| Main income: social transfers | 0.880^***^ | -0.0904 | 0.691^***^ | -0.0580 | 0.577^**^ | 0.197 | 0.359^*^ | 0.0275 |
| Main income: old-age benefits | Ref. cat. | Ref. cat. | Ref. cat. | Ref. cat. | Ref. cat. | Ref. cat. | Ref. cat. | Ref. cat. |
| Main income: other private | 1.005^***^ | 0.0891 | 0.895^***^ | -0.167 | -1.352^***^ | -1.016^***^ | -1.850^***^ | -1.916^*^ |
| Total no. of different income components | -0.104^**^ | -0.113^**^ | -0.0572 | -0.110^**^ | -0.0680^**^ | -0.0446^*^ | -0.0340 | -0.0572^*^ |
| Activity status^2^: full time work | Ref. cat. | Ref. cat. | Ref. cat. | Ref. cat. | Ref. cat. | Ref. cat. | Ref. cat. | Ref. cat. |
| Activity status: part time work | 0.260 | -0.300^**^ | -0.0218 | -0.0433 | 0.166^*^ | 0.155^*^ | 0.0864 | 0.245^***^ |
| Activity status: unemployed | -0.341 | -0.453^***^ | -0.540^***^ | -0.505^**^ | 0.332^*^ | 0.597^***^ | 0.362^***^ | 0.468^***^ |
| Activity status: retired | 0.0740 | -0.286^*^ | -0.0646 | -0.369 | 0.0120 | 0.0620 | 0.0344 | 0.0431 |
| Activity status: student, other | -0.338 | -0.450 | -0.601^***^ | -0.774^***^ | 0.375 | 0.430^***^ | 0.401^**^ | 0.638^***^ |
| Activity status: housework | -0.206 | -0.310^*^ | -0.189 | -0.408^*^ | 0.316^***^ | 0.435^***^ | 0.386^***^ | 0.410^***^ |
| No. hh members >15 with >1 employment | 0.257 | 0.0375 | -0.0671 | -0.0279 | -0.122 | -0.147 | -0.305^***^ | -0.166^*^ |
| Employ. status changes: none | Ref. cat. | Ref. cat. | Ref. cat. | Ref. cat. | Ref. cat. | Ref. cat. | Ref. cat. | Ref. cat. |
| Employ. status changes: 1 | -0.0948 | 0.158 | 0.0602 | -0.118 | 0.210^**^ | 0.137^*^ | 0.133^*^ | 0.153^*^ |
| Employ. status changes: >1 | -0.0702 | 0.135 | 0.0606 | -0.0870 | 0.309^*^ | 0.0343 | 0.130 | 0.199 |
| Age | 0.00689 | -0.0101 | 0.00428 | -0.00440 | -0.0374^***^ | -0.00596^**^ | -0.00283 | 0.0403^***^ |
| Age squared |  | 0.000189^*^ |  |  | 0.000332^***^ |  |  | -0.000459^***^ |
| Male | Ref. cat. | Ref. cat. | Ref. cat. | Ref. cat. | Ref. cat. | Ref. cat. | Ref. cat. | Ref. cat. |
| Female | -0.147 | -0.00452 | 0.0126 | -0.0902 | -0.0601 | -0.0526 | 0.0186 | -0.0914 |
| Household sickness (Median)^3^ | -0.0344 | 0.00278 | -0.0489 | -0.0314 | -0.00714 | -0.000140 | 0.0323 | 0.0645 |
| Education: basic | Ref. cat. | Ref. cat. | Ref. cat. | Ref. cat. | Ref. cat. | Ref. cat. | Ref. cat. | Ref. cat. |
| Education: middle | 0.230^*^ | 0.355^***^ | 0.169 | 0.366^**^ | -0.282^***^ | -0.200^***^ | -0.0568 | -0.120 |
| Education: high | 0.453^***^ | 0.463^***^ | 0.271^*^ | 0.444^**^ | -0.387^***^ | -0.401^***^ | -0.250^**^ | -0.197^*^ |
| Education: specialized | 0.913^***^ | 0.752^***^ | 0.524^***^ | 0.836^***^ | -0.468^***^ | -0.426^***^ | -0.269^**^ | -0.370^***^ |
| Retired household | 0.376 | -0.392 | 0.297 | -0.811 | 0.123 | 0.142 | 0.147 | -0.288 |
| Single HH, not retired | -0.162 | -0.0149 | -0.296^*^ | -0.280 | -0.0838 | 0.0887 | 0.109 | 0.0698 |
| MPH, no children | Ref. cat. | Ref. cat. | Ref. cat. | Ref. cat. | Ref. cat. | Ref. cat. | Ref. cat. | Ref. cat. |
| Single parent | -0.298 | -0.363 | -0.518^*^ | -0.615^**^ | -0.126 | 0.354^**^ | 0.100 | 0.155 |
| MPH, children | -0.118 | -0.232^**^ | -0.462^***^ | -0.363^**^ | 0.0956 | 0.184^**^ | 0.191^***^ | 0.197^**^ |
| Region: Vienna (capital, >1,000,000 inh.) | Ref. cat. | Ref. cat. | Ref. cat. | Ref. cat. | Ref. cat. | Ref. cat. | Ref. cat. | Ref. cat. |
| >100,000 inhabitants | -0.131 | -0.159 | -0.0163 | 0.0330 | -0.0683 | 0.0374 | 0.0997 | 0.0292 |
| >10,000 inhabitants | -0.0232 | -0.0354 | -0.0682 | -0.0296 | -0.0207 | 0.0790 | 0.00519 | 0.0607 |
| <=10,000 inhabitants | 0.00797 | -0.123 | -0.202^**^ | -0.102 | -0.0812 | 0.0169 | 0.0983^*^ | -0.00606 |
| CAPI | Ref. cat. | Ref. cat. | Ref. cat. | Ref. cat. | Ref. cat. | Ref. cat. | Ref. cat. | Ref. cat. |
| CATI | 0.00881 | 0.0672 | 0.0231 | 0.00767 | -0.125^*^ | -0.0680 | -0.0430 | -0.160^**^ |
| Sum of proxy interviews in household | 0.0168 | 0.0377 | -0.00393 | -0.0219 | -0.00408 | 0.0152 | 0.0258 | 0.0963^**^ |
| Interview month | 0.0299 | 0.0236 | -0.0443 | -0.000144 | 0.0158 | 0.0184 | 0.0182 | -0.0325 |
| SILC round 1 | Ref. cat. | Ref. cat. | Ref. cat. | Ref. cat. | Ref. cat. | Ref. cat. | Ref. cat. | Ref. cat. |
| SILC round 2 | 0.0526 | -0.153 | -0.0235 | -0.0285 | -0.0707 | 0.0272 | -0.115^*^ | 0.0890 |
| SILC round 3 | -0.110 | -0.147 | -0.438^***^ | -0.169 | 0.0828 | 0.00924 | 0.00418 | 0.0941 |
| SILC round 4 | -0.126 | -0.160 | -0.237^*^ | -0.319^**^ | 0.0439 | 0.137 | -0.127^*^ | 0.0871 |
| Constant | 8.443^***^ | 9.999^***^ | 10.29^***^ | 11.14^***^ | -8.519^***^ | -9.770^***^ | -10.20^***^ | -10.36^***^ |
| N (Households) | 2450 | 2503 | 2448 | 2264 | 3098 | 3238 | 3546 | 3192 |

Note: Poisson regression models. Standard errors (not displayed) account for complex survey design (strata=federal states). Epinc: Equivalised household income.


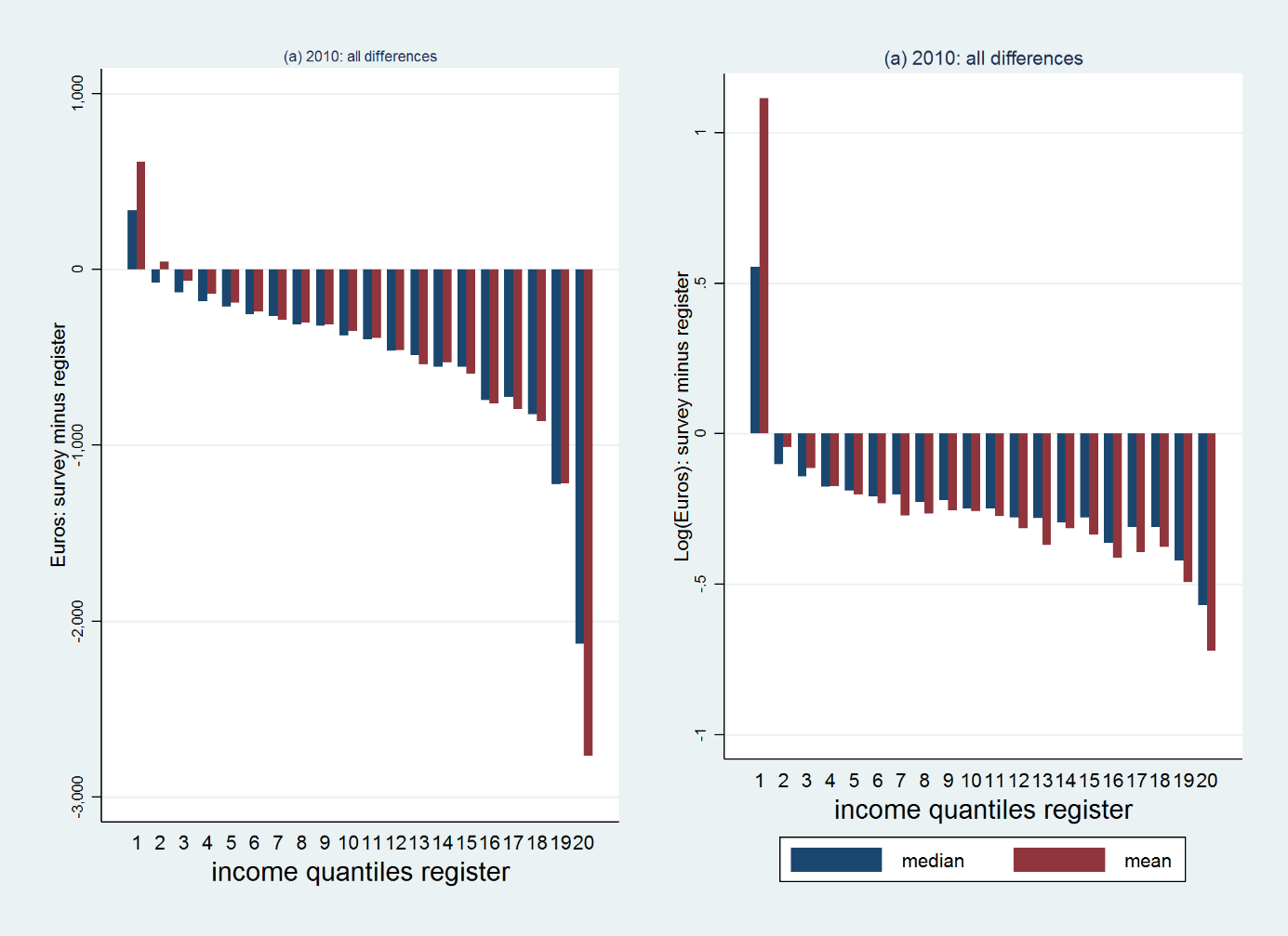


Figure A 5 Median and mean of absolute (left) and log(deviation) (right) for equivalised household income for 20 quantiles derived from registers. Weighted data. Persons are units of observation. Income quantiles based on register data. Difference = survey minus register. For logarithmic deviations see Figure A 4.

Table A 10: Cross-sectional regression models (2010), dependent variable derived from single question

|  | **Over-reporting (survey > register)** | | | **Under-reporting (survey < register)** | | |
| --- | --- | --- | --- | --- | --- | --- |
|  | **(1)**  **Mlogit** | **(2)**  **OLS** | **(3)**  **OLS (dep. var in logs)** | **(4)**  **Mlogit** | **(5)**  **OLS** | **(6)**  **OLS**  **(dep. var in logs)** |
| Ln(Epinc) | 0.210^***^ |  | -0.122^**^ | 5.952^***^ |  | 1.611^***^ |
| Epinc |  | -0.0679^***^ |  |  | 0.0366^***^ |  |
| Epinc squared |  | 0.00000113^***^ |  |  | 0.000000146^***^ |  |
| Satisfaction with household income (median)^1^ | 1.355^***^ | 124.0^***^ | 0.118^***^ | 0.816^***^ | -90.95^***^ | -0.0786^***^ |
| Main income: employed | 0.794 | -125.6 | -0.209 | 0.463 | -82.63 | -0.435^***^ |
| Main income: self-employment | 0.949 | -275.5 | 0.157 | 0.237^*^ | -54.41 | -0.551^***^ |
| Main income: social transfers | 0.976 | -139.9 | -0.407^*^ | 0.766 | -75.63 | -0.183 |
| Main income: old-age benefits | Ref. cat. | Ref. cat. | Ref. cat. | Ref. cat. | Ref. cat. | Ref. cat. |
| Main income: other private | 0.400 | 251.0 | 0.448 | 0.246 | 209.4^*^ | -0.418 |
| Total no. of different income components | 0.991 | -31.54 | 0.0675^**^ | 1.061 | 10.92 | 0.0516^***^ |
| Activity status^2^: full time work | Ref. cat. | Ref. cat. | Ref. cat. | Ref. cat. | Ref. cat. | Ref. cat. |
| Activity status: part time work | 0.821 | -147.6^*^ | -0.173 | 1.040 | 17.83 | 0.0760 |
| Activity status: unemployed | 0.956 | -286.8^***^ | -0.243 | 1.315 | 57.47 | -0.0289 |
| Activity status: retired | 0.973 | -197.6 | -0.151 | 1.310 | 74.32^*^ | -0.0354 |
| Activity status: student, other | 0.467 | -491.5^***^ | -0.895^***^ | 0.570 | 95.87 | 0.300^*^ |
| Activity status: housework | 1.528 | -233.0^**^ | -0.233^*^ | 1.946^*^ | 122.3^***^ | 0.226^***^ |
| No. hh members >15 with >1 employment | 1.217 | 39.12 | 0.153 | 1.061 | -0.274 | -0.0110 |
| Employ. status changes: none | Ref. cat. | Ref. cat. | Ref. cat. | Ref. cat. | Ref. cat. | Ref. cat. |
| Employ. status changes: 1 | 1.367 | -24.74 | 0.0411 | 0.914 | 93.77^***^ | 0.0211 |
| Employ. status changes: >1 | 4.473^**^ | 49.59 | 0.123 | 2.128 | 175.5^***^ | 0.218^*^ |
| Age | 0.994 | 2.141 | 0.00776^**^ | 0.994 | -0.240 | 0.00671 |
| Age squared | 1.000 |  |  | 1.000 |  | -0.0000594 |
| Male | Ref. cat. | Ref. cat. | Ref. cat. | Ref. cat. | Ref. cat. | Ref. cat. |
| Female | 0.769 | -3.269 | -0.0784 | 0.873 | 2.635 | -0.0321 |
| Household sickness (Median)^3^ | 1.023 | 36.14 | 0.00690 | 1.052 | -3.631 | -0.0155 |
| Education: basic | Ref. cat. | Ref. cat. | Ref. cat. | Ref. cat. | Ref. cat. | Ref. cat. |
| Education: middle | 1.282 | 166.3^***^ | 0.151^*^ | 0.656 | -62.65^***^ | 0.0413 |
| Education: high | 2.197^**^ | 236.4^***^ | 0.355^***^ | 0.761 | -137.2^***^ | 0.0170 |
| Education: specialized | 2.019^*^ | 461.0^***^ | 0.528^***^ | 0.393^***^ | -302.9^***^ | -0.122 |
| Retired household | 0.649 | -250.4 | -0.870^***^ | 0.878 | -57.99 | -0.446^***^ |
| Single HH, not retired | 0.762 | -157.9^*^ | -0.717^***^ | 1.047 | 2.961 | -0.475^***^ |
| MPH, no children | Ref. cat. | Ref. cat. | Ref. cat. | Ref. cat. | Ref. cat. | Ref. cat. |
| Single parent | 0.969 | -224.5^**^ | -0.535^***^ | 2.521^*^ | 85.87^*^ | 0.196^*^ |
| MPH, children | 0.564^*^ | -113.7 | -0.132 | 1.885^**^ | 118.2^***^ | 0.391^***^ |
| Region: Vienna (capital, >1,000,000 inh.) | Ref. cat. | Ref. cat. | Ref. cat. | Ref. cat. | Ref. cat. | Ref. cat. |
| >100,000 inhabitants | 0.943 | -53.50 | -0.0312 | 1.024 | 43.50 | 0.0921 |
| >10,000 inhabitants | 0.786 | -45.47 | 0.170^*^ | 1.046 | 39.07 | 0.0466 |
| <=10,000 inhabitants | 0.934 | -20.78 | 0.0533 | 1.033 | 35.27 | 0.149^***^ |
| CAPI | Ref. cat. | Ref. cat. | Ref. cat. | Ref. cat. | Ref. cat. | Ref. cat. |
| CATI | 1.013 | 64.09 | 0.161^*^ | 0.934 | -27.10 | -0.137^**^ |
| Sum of proxy interviews in hh | 1.175 | -71.67^*^ | 0.130^**^ | 1.140 | 29.08^*^ | 0.178^***^ |
| Interview month | 1.072 | -5.927 | 0.0153 | 0.949 | -2.955 | -0.00835 |
| SILC round 1 | Ref. cat. | Ref. cat. | Ref. cat. | Ref. cat. | Ref. cat. | Ref. cat. |
| SILC round 2 | 1.244 | -36.57 | -0.0251 | 1.091 | -46.77^*^ | -0.0508 |
| SILC round 3 | 0.763 | -155.3^**^ | -0.230^**^ | 0.854 | -33.39 | -0.0119 |
| SILC round 4 | 1.111 | -34.81 | -0.0871 | 1.087 | -3.899 | 0.0168 |
| Constant |  | 751.6^*^ | 6.373^***^ |  | 60.79 | -9.260^***^ |
| R^2^ (OLS models) |  | 0.345 | 0.214 |  | 0.796 | 0.545 |
| N (Households) | 6074 | 954 | 2397 | 6074 | 5001 | 3491 |

Note: * p < 0.05, ** p < 0.01, *** p < 0.001. ^1^ scale from 1 to 6; 1=very unhappy, 6= very happy. ^2^ Self-reported labor status in income reference period for 2010 for more than 6 months. ^3^ scale from 1 to 5; 1=very good 5 = very bad. Dep. var. = [equivalised monthly household income self-reported with a single question] minus [1/12*epinc_register_]

*Multinomial logit regression models* using sampling weights: Coefficients of the model are estimated at once. Sample size in col. (1) and (4) refers to the sum over all 3 categories of the dependent variable. Coefficients show odds ratios. Odds For the dependent variable, the reference category refers to households with a difference between equivalised household incomes that lies within the range of +/- 5%. Standard errors (not displayed) account for complex stratified survey design. Pseudo R^2^ measures are not available for Maximum Likelihood estimation as the assumption of observations being independent and identically distributed (*iid*) is not fulfilled. MPH: Multiple person household.

*OLS regression models* using sampling weights: Dependent variable is epincdelta (survey minus register). Standard errors (not displayed) account for complex survey design (strata=federal states).

Table A 11: Results from panel regression models (4 rounds 2008 to 2011) with household fixed effects, dependent variable derived from single question

| Dep. var. = [equivalised monthly household income self-reported with a single question] minus [1/12*epinc_register_] | **Levels** |  | **Logs** |  |
| --- | --- | --- | --- | --- |
|  | **Unbalanced** | **Balanced** | **Unbalanced** | **Balanced** |
| Equivalised annual income: Lowest 5% | 46.85 | -186.6^*^ | -0.175 | -0.823^***^ |
| 10% percentile | -210.1^***^ | -369.8^***^ | -0.788^***^ | -1.232^***^ |
| 15% percentile | -260.5^***^ | -395.7^***^ | -0.725^***^ | -1.245^***^ |
| 20% percentile | -230.4^***^ | -310.8^***^ | -0.561^***^ | -0.802^***^ |
| 25% percentile | -218.9^***^ | -257.2^***^ | -0.537^***^ | -0.697^***^ |
| 30% percentile | -160.9^***^ | -261.7^***^ | -0.397^***^ | -0.543^***^ |
| 35% percentile | -120.9^***^ | -158.0^***^ | -0.319^***^ | -0.359^***^ |
| 40% percentile | -94.49^***^ | -169.3^***^ | -0.258^***^ | -0.491^***^ |
| 45% percentile | -44.92^*^ | -107.1^*^ | -0.115^*^ | -0.153 |
| 50% percentile | Ref. cat. | Ref. cat. | Ref. cat. | Ref. cat. |
| 55% percentile | 65.99^**^ | 63.49 | 0.169^***^ | 0.101 |
| 60% percentile | 102.7^***^ | 61.13 | 0.212^***^ | 0.0702 |
| 65% percentile | 149.1^***^ | 76.07 | 0.284^***^ | 0.119 |
| 70% percentile | 262.0^***^ | 183.1^***^ | 0.445^***^ | 0.291^**^ |
| 75% percentile | 334.0^***^ | 283.9^***^ | 0.562^***^ | 0.375^***^ |
| 80% percentile | 484.2^***^ | 451.9^***^ | 0.766^***^ | 0.632^***^ |
| 85% percentile | 616.1^***^ | 532.8^***^ | 0.897^***^ | 0.770^***^ |
| 90% percentile | 816.3^***^ | 722.1^***^ | 1.146^***^ | 0.986^***^ |
| 95% percentile | 1203.3^***^ | 1055.9^***^ | 1.390^***^ | 1.185^***^ |
| Top 5% | 2525.9^***^ | 2201.3^***^ | 2.008^***^ | 1.922^***^ |
| HH Satisfaction w. household income (Median)^1^ | -11.25 | -0.677 | -0.0200 | 0.00590 |
| Main income: employed | -90.27 | -345.8 | -0.160 | -0.348 |
| Main income: self-employment | -176.7 | -245.2 | -0.289^*^ | -0.393 |
| Main income: social transfers | -121.3 | -285.7 | -0.144 | -0.206 |
| Main income: old-age benefits | Ref. cat. | Ref. cat. | Ref. cat. | Ref. cat. |
| Main income: other | 215.6 | 176.1 | 0.236 | 0.269 |
| Total no. of different income components | 6.805 | -2.828 | 0.0372^***^ | 0.0337 |
| Activity status^2^: Full Time | Ref. cat. | Ref. cat. | Ref. cat. | Ref. cat. |
| Activity status: Part Time | 16.89 | -35.67 | -0.0165 | 0.0489 |
| Activity status: Unemployed | -44.64 | -60.16 | -0.165^*^ | -0.204 |
| Activity status: Retired | 34.62 | 43.23 | 0.00321 | -0.0255 |
| Activity status: Student, School, other | -15.51 | -51.63 | -0.0849 | -0.0547 |
| Activity status: Housework | -32.08 | 67.61 | -0.115^*^ | 0.0418 |
| No. hh members >15 with >1 employment | 23.66 | 69.10 | -0.0368 | 0.0898 |
| 0 employment status changes | Ref. cat. | Ref. cat. | Ref. cat. | Ref. cat. |
| 1 employment status change | 26.72^*^ | 50.21 | 0.0599^*^ | 0.0295 |
| >1 employment status changes | 42.56 | 34.46 | 0.145^*^ | 0.0644 |
| Household sickness (Median)^3^ | 16.02 | 27.75 | 0.000805 | 0.0313 |
| Education: basic | Ref. cat. | Ref. cat. | Ref. cat. | Ref. cat. |
| Education: middle | 1.861 | 35.78 | 0.0639 | 0.0784 |
| Education: high | 4.794 | 36.60 | 0.0646 | 0.0614 |
| Education: specialized | -70.65 | 20.89 | 0.118 | 0.205 |
| Retired household | -215.3 | -344.2 | -0.448^***^ | -0.536^*^ |
| Single household, not retired | -13.74 | -48.71 | -0.353^***^ | -0.338^***^ |
| Multiple person household no children | Ref. cat. | Ref. cat. | Ref. cat. | Ref. cat. |
| Single parent household | 143.0^**^ | 165.8 | 0.231^*^ | 0.499^**^ |
| Multiple person household w. children | 125.3^***^ | 107.1 | 0.301^***^ | 0.359^***^ |
| Region: Vienna (capital city, >1,000,000 inh.) | Ref. cat. | Ref. cat. | Ref. cat. | Ref. cat. |
| >100,000 inhab. | 414.0^*^ | 486.1 | 0.466^*^ | 0.818^*^ |
| >10,000 inhab. | 202.9 | 92.53 | 0.303 | 0.0873 |
| <=10,000 inhab. | 147.4 | 176.4 | 0.164 | 0.582^**^ |
| CAPI | Ref. cat. | Ref. cat. | Ref. cat. | Ref. cat. |
| CATI | -10.54 | -1.978 | 0.0323 | 0.0739 |
| Sum of proxy interviews in household | 11.18 | -0.588 | 0.0556^**^ | 0.0289 |
| Interview month | -0.692 | 5.162 | 0.00158 | 0.0109 |
| SILC round 1 | Ref. cat. | Ref. cat. | Ref. cat. | Ref. cat. |
| SILC round 2 | 30.46^*^ | 79.72^**^ | 0.0191 | 0.0309 |
| SILC round 3 | 32.82^*^ | 45.42 | 0.0291 | 0.00451 |
| SILC round 4 | 89.68^***^ | 96.84^**^ | 0.103^***^ | 0.0698 |
| N (Households) | 23141 | 4312 | 23101 | 4306 |

Note: * p < 0.05, ** p < 0.01, *** p < 0.001. OLS regression models with fixed effects for households. Unweighted estimates. Socio-demographic variables with low within-variation were not included in the model. Estimates for unbalanced panel (2008-2011) and 4 wave balanced panel sample (2008-2011). ^1^ scale from 1 to 6; 1=very unhappy, 6= very happy. ^2^ Self-reported labor status in income reference period for 2010 for more than 6 months. ^3^ scale from 1 to 5; 1=very good 5 = very bad. HH… Household.
